# Synthesis, structure-activity relationships and biological evaluation of benzimidazole derived sulfonylurea analogues as a new class of antagonists of P2Y1 receptor

**DOI:** 10.3389/fphar.2023.1217315

**Published:** 2023-05-26

**Authors:** Sehrish Bano, Zahid Hussain, Peter Langer, Gary A. Weisman, Jamshed Iqbal

**Affiliations:** ^1^ Centre for Advanced Drug Research, COMSATS University Islamabad, Abbottabad Campus, Abbottabad, Pakistan; ^2^ Department of Pharmacy, COMSATS University Islamabad, Abbottabad, Pakistan; ^3^ Institut für Chemie, Universität Rostock, Rostock, Germany; ^4^ Department of Biochemistry, University of Missouri-Columbia, Columbia, MO, United States

**Keywords:** purinergic receptors, benzimidazole, calcium signalling, expression analysis, docking studies

## Abstract

The P2Y receptors are responsible for the regulation of various physiological processes including neurotransmission and inflammatory responses. These receptors are also considered as novel potential therapeutic targets for prevention and treatment of thrombosis, neurological disorders, pain, cardiac diseases and cancer. Previously, number of P2Y receptor antagonists has been investigated but they are less potent and non-selective with poor solubility profile. Herein, we present the synthesis of new class of benzimidazole derived sulfonylureas (**1a-y**) as potent antagonists of P2Y receptors, with the specific aim to explore selective antagonists of P2Y1 receptors. The efficacy and selectivity of the synthesized derivatives **1**) against four P2Y receptors i.e., t-P2Y1, h-P2Y2, h-P2Y4, and r-P2Y6Rs was carried out by calcium mobilization assay. The results revealed that except **1b, 1d, 1l, 1m, 1o, 1u, 1v, 1w,** and **1y**, rest of the synthesized derivatives exhibited moderate to excellent inhibitory potential against P2Y1 receptors. Among the potent antagonists, derivative **1h** depicted the maximum inhibition of P2Y1 receptor in calcium signalling assay, with an IC_50_ value of 0.19 ± 0.04 µM. The potential of inhibition was validated by computational investigations where bonding and non-bonding interactions between ligand and targeted receptor further strengthen the study. The best identified derivative **1h** revealed the same binding mechanism as that of already reported selective antagonist of P2Y1 receptor i.e (1-(2- (2-tert-butyl-phenoxy) pyridin-3-yl)-3–4-(trifluoromethoxy) phenylurea but the newly synthesized derivative exhibited better solubility profile. Hence, this derivative can be used as lead candidate for the synthesis of more potential antagonist with much better solubility profile and medicinal importance.

## 1 Introduction

Different cellular activities are controlled by extracellular nucleotides through P2X which is ionotropic and P2Y which is G protein-coupled receptor i.e., endocrine and exocrine secretion, neurotransmission, immune cell regulation, proliferation, differentiation and intracellular signalling (Burnstock, 2017). Eight types of human P2Y receptors have been characterized so far which were further divided into two distinct subgroups on basis of phylogenetic structure (Xu et al., 2018). The first group couples to Gq protein and contains five receptors i.e., ADP (P2Y1), ATP, UTP (P2Y2), ATP (P2Y11), UTP (P2Y4) and UDP (P2Y6) while second group couples to Gi protein includes P2Y12-14Rs (Hillmann et al., 2009).

P2YRs are expressed in almost all human tissues and exerts various biological functions (Uddin et al., 2016). Over-expression of P2Y receptors have been found in different pathological conditions and thus considered as therapeutic targets for several diseases such as neurodegenerative disorders thrombosis, pain, cardiovascular diseases and cancer (Burnstock, 2006; Hechler and Gachet, 2015; Alves et al., 2018). Some antithrombotic drugs such as clopidogrel, prasugrel and ticagrelor attain their result by inhibiting the effects of human P2Y12 receptor and are present in the market (Jacobson et al., 2009).

The N-terminus which is extracellular of P2Y receptor has many glycosylation sites (N-linked), and it has seven transmembrane spanning motifs that help in the creation of the ligand pocket (Erb and Weisman, 2012). Additionally, there are three intracellular and extracellular loops which are helpful in coupling with G-proteins, as well as an intracellular C-terminus containing a few phosphorylation positions which causes activation of protein kinases (Parravicini et al., 2008; Gacasan et al., 2017). Purinergic receptors express the distinctive feature that on stimulation of many of the P2Y subtypes, they respond by causing a quick change in signals of intracellular [Ca^+2^] which is useful for the screening of agonist and antagonist of purinergic receptors by performing calcium flux assay in P2Y transfected 1321N1 astrocytoma cell line (Govindan and Taylor, 2012). Cell line such as; 1321N1 astrocytoma cell line has been widely used for the characterization of receptor systems due to absence of an inherent nucleotide receptor and abundance of transduction elements. This allows the receptors which are transfected to connect to the host cell’s signalling pathway (Filtz et al., 1994).

Epithelial cells, macrophages, neutrophils and cardiomyocytes expressed P2Y2 and P2Y4Rs. These receptors are also expressed in some organs such as the heart, kidneys, brain, liver and spleen (Li et al., 2013; Chen et al., 2019). P2Y2 and P2Y4Rs antagonists play key role in prevention of inflammation and tumour (Rafehi et al., 2017). Widespread expression of the P2Y6 receptor is associated with inflammatory reactions and is thought to be a key target for inflammatory illnesses (Anwar et al., 2020).

**FIGURE 1:**
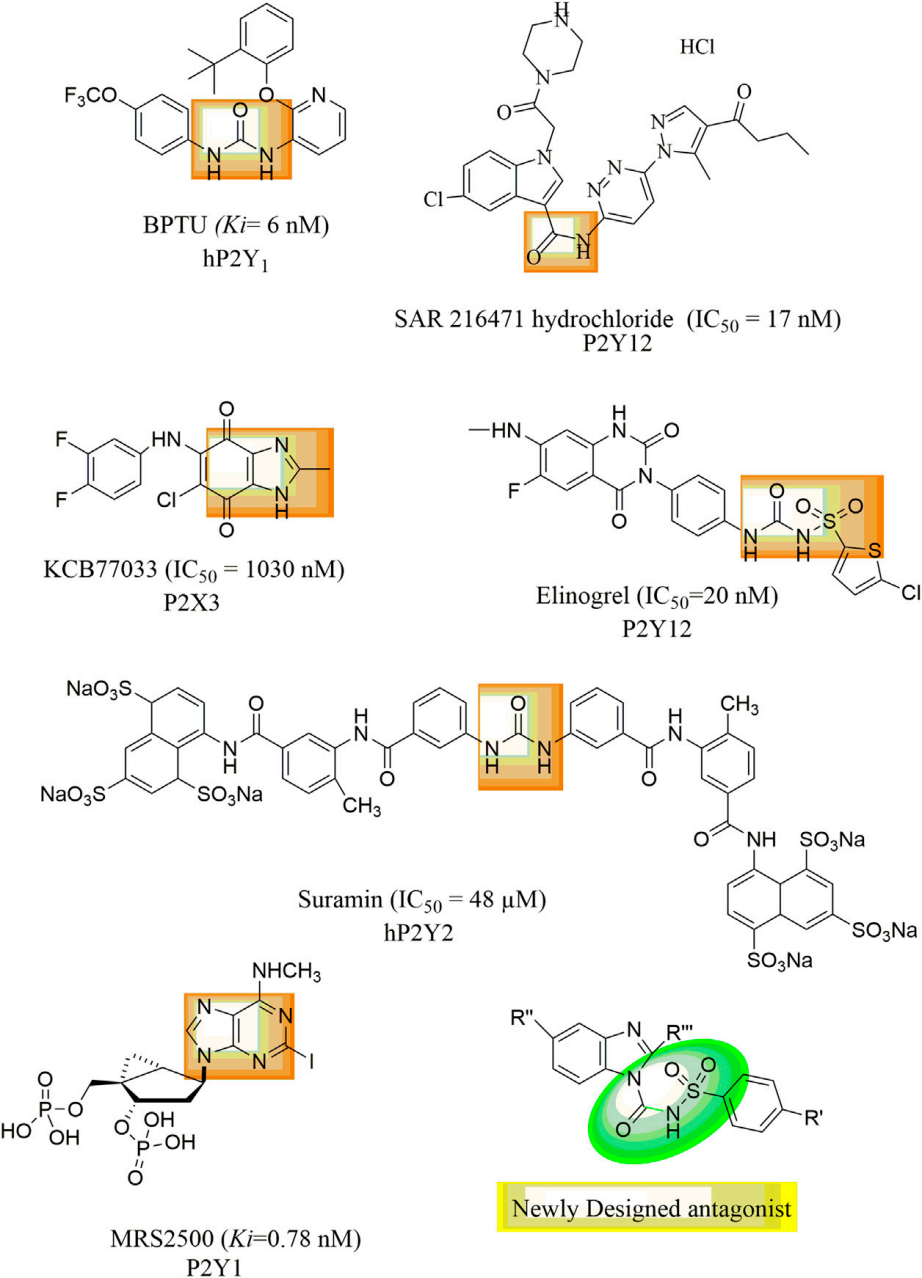
Reported antagonist of P2Y receptor.

P2Y1 receptors have been discovered and reported in humans, rats, mice, cows, chickens, turkeys, and *Xenopus* (Abbracchio et al., 2006). P2Y1 receptors have been identified as a potential therapy option for thrombosis because they are found in the placenta, platelets, brain, and prostate (Pi et al., 2013). Compared to ATP, ADP is a stronger P2Y1 receptor agonist. According to studies, the P2Y1 receptor’s extracellular loops need to have four cysteine residues in order to properly travel to the cell surface (Hoffmann et al., 1999). The P2Y1 receptor binds to Gα11 Gαq, and its activation raises intracellular IP3 levels via PLC, releasing Ca^2+^ from intracellular reserves in a way that is PTX-insensitive. P2Y1R generates a quick and transient increase in calcium intracellular signal which causes change in platelet shape and initiates the platelet activation process. In addition to platelet aggregation, P2Y1 receptors are also active in many other physiological processes i.e., vascular inflammation, Ca^2+^ transmission and stimulation of extracellular signals which controlled kinase in glial cells (Hechler and Gachet, 2011). BPTU (1-(2- (2-tert-butyl-phenoxy) pyridin-3-yl)-3–4-(trifluoromethoxy) phenylurea is selective P2Y1 inhibitor and its different urea isosteres i.e., aminooxazole, aminoimidazole and aminothiazole are reported. However, it showed low aqueous solubility, high lipophilicity, and strong binding to human proteins (Ruel et al., 2013). Most of the molecules in urea series had submicromolar potencies, but in general the urea compounds had poor solubility and low metabolic stability in microsomes of liver (Bach et al., 2013). If the urea functionality was replaced with a sulfonylurea group, good binding affinity and increased aqueous solubility can be expected. Therefore, there is need for development and synthesis of P2Y1R antagonists with enhanced pharmaceutical features. Elinogrel,SAR216471, Suramin, Reactive Blue-2, Butylphenoxy-(trifluoromethoxy) phenylurea (BPTU), MRS2500 and Pyridoxal-phosphate-6-azophenyl-2′,4′-disulfonic acid [PPADS] are some stated inhibitors of P2YRs as shown in Figure 1 (Oestreich, 2010; Boldron et al., 2014; Peng et al., 2018; Bano et al., 2021). Recently, Benzimidazole-4,7-dione-based analogues were reported as a new class of compounds for the development of P2X3R antagonists (Bae et al., 2022). In the proposed study a new series of benzimidazole derived sulfonylureas were synthesized as potent antagonist of P2Y receptor subtype (P2Y1). For this purpose, stably transfected t-P2Y1, h-P2Y2, h-P2Y4, and r-P2Y6-1321N1 astrocytoma cell line was used and fluorescent based Ca^2+^ influx was assessed by using a Ca^2+^ binding dye i.e., Fura-2 a.m. The cytotoxicity component was removed using a cell viability assay (MTT assay). In order to confirm the *in silico* interaction between target and ligand, *in silico* studies were conducted.

## 2 Materials and methods

### 2.1 Reagents, solvents and chemicals

Unless otherwise stated, all of the reagents and chemicals were obtained from the commercial sources (Sigma-Aldrich, Alfa Aesar and Merck) and were used without any additional purification. Using capillary tubes (open ended) and the Gallenkamp melting point equipment, melting points of synthetic substances were calculated. On a BRUKER spectrometer, NMR spectroscopy (Proton and carbon NMR) was carried out in both DMSO and chloroform. FTIR (Agilent Technologies’ Cary 630) spectrophotometer was employed to conduct IR spectrum research. LCMS Agilent 1,200/6,210 (TOF) was used for the HRMS analysis, and the electrospray ionisation (ESI) or electron ionisation (EI, 70 eV) methods were used to get the HRMS data.

### 2.2 Synthesis of benzimidazole derived sulfonylurea analogues

#### 2.2.1 General procedure for synthesis of aryl sulphonamides (4a-h)

Ammonium hydroxide 3 (33% solution, 10 mL) was added to suitable (**2a-h**) Sulfonyl chlorides (0.2 mmol) in 10 mL of methanol 10 mL. For 2 hours, the mixture of reaction was allowed to stir continuously at rt. TLC verified that reaction had finished. Then this reaction mixture was dried by evaporating it under decreased pressure. Product was then extracted with the 15 mL each of H_2_O and 15 mL CH_3_COOC_2_H_5_. To extract the compounds as solid-products, layer of Ethyl acetate was isolated, and washed with saturated saline thrice with 15 mL and at the end product was dried out with anhydrous Na_2_SO_4_, and further and dried up by evaporation (Ullah et al., 2021).

#### 2.2.2 Method for synthesizing carbamate intermediates (7a-f)

Anhydrous Tetrahydrofuran (5 mL) was used and chilled a combination of compound **5a-f** (1 mmol) and Triethylamine (2 mL) to 0°C by using ice-bath. Thereto, slowly emulsifying Phenyl chloroformate 6 (222 mg, 2 mmol) solution in a THF (5 mL) which is anhydrous was then added to it. For a further 2hrs, this mixture remained continued for stirring at RT. After this, the mixture of reaction was vacuum evaporated, 30 mL of Ethyl acetate was used for extracting the product, and then washed away with 30 mL of saline. Using anhydrous Na_2_SO_4_, the organic upper layer was isolated and dried. It was then filtered and evaporated for dryness. Without additional purification, these intermediates were employed in the last stage after being verified by TLC.

#### 2.2.3 Procedure for synthesis of the sulfonyl urea products (1a-y)

Compounds **4a-h** (0.1 mmol), anhydrous Sodium hydride (0.5 mmol, 69 mg) and **7a-f** (0.12 mmol) intermediates were combined and allowed for stirring in dry Dimethylformamide (5 mL) at RT for a whole day. Then the mixture of reaction was washed away with 20 mL of H_2_O after the reaction was finished, and the upper organic portion was then isolated, recovered with the anhydrous Na_2_SO_4_, filtrated, and allowed for evaporation until it get dried. Recrystallization was used to purify the residual residue.

##### 2.2.3.1 2-Amino-N-((3,5-dichloro-2-hydroxyphenyl) sulfonyl)-5-nitro-1H- benzo [d]imidazole-1-carboxamide (1a)

Yield: 65%; off-white powder; mp: 107–110°C; FTIR (ῡ, cm-1): 2,939 (N–H), 3,450 (OH), 1,613 (C=O), ^1^H NMR (DMSO-d6 - 250 MHz) δ (ppm) 10.81 (s, 1H, NH), 10.11 (s, 1H, OH), 7.85 (d, J = 1.51 Hz, 1H, Ar-H), 7.54 (m, 2H, Ar-H), 7.38 (m, 2H, Ar-H), 7.37 (s, 2H, NH_2_). ^13^C NMR (63 MHz, DMSO-d6) δ 168.7 (C=O), 167.9 (C-OH), 148.4 (C-NH_2_), 133.8 (C-NO_2_), 132.9, 130.2, 125.2 (C-Cl), 122.0, 121.3, 111.8, 104.7; HRMS (ESI-TOF) m/z: calcd [M+H]^+^ for C_14_H_9_Cl_2_N_5_O_6_S, 444.1270; found, 444.1265.

##### 2.2.3.2 2-Amino-5-nitro-N-tosyl-1H-benzo [d]imidazole-1-carboxamide (Ib)

Yield: 62%; light brown solid; mp: 113–115°C; FTIR (ῡ, cm-1): 3,429 (N–H), 1,633 (C=O), 1,104 (S=O), ^1^H NMR (DMSO-d6 -250 MHz) δ 10.76 (s, 1H, NH), 7.54 (d, J = 1.62 Hz, 2H), 7.51 (dd, J = 7.37, 1.45 Hz, 3H, Ar-H), 6.87 (s, 2H, NH_2_), 6.83 (m, 2H, Ar-H), 1.95 (s, 3H, CH_3_).^13^C NMR (63 MHz, DMSO-d6) δ 168.7 (C=O), 159.2 (C-NH_2_), 156.5, 150.5, 140.5, 138.0, 127.0, 126.9, 121.9, 115.3, 112.7, 111.2 (Ar-Cs), 20.0 (C-CH3); HRMS (ESI-TOF) m/z: calcd [M+H]^+^ for C_15_H_13_N_5_O_5_S, 375.0637; found, 375.0636.

##### 2.2.3.3 2-Amino-N-((2-methyl-5-nitrophenyl)sulfonyl)-5-nitro-1H-benzo [d]imidazole-1- carboxamide (Ic)

Yield: 77%; off-white powder; mp: 117–119°C; FTIR (ῡ, cm-1): 3,262 (N–H), 1,633 (C=O), 1,034 (S=O), ^1^H NMR (DMSO-d6 -250 MHz) δ 10.68 (s, 1H, NH), 8.86 (m, 2H, Ar-H), 8.09 (dd, J = 7.52, 1.46 Hz, 1H, Ar-H), 8.05 (m, 2H, Ar-H), 7.47 (s, 2H, NH_2_), 7.43 (d, J = 7.47 Hz, 1H, Ar-H), 1.94 (s, 3H, CH_3_).^13^C NMR (63 MHz, DMSO-d6) δ 167.9 (C=O), 147.6, 144.8, 144.2, 132.1, 127.2, 123.2, 121.1, 20.1(C-CH_3_); HRMS (ESI-TOF) m/z: calcd [M+H]^+^ for C_15_H_12_N_6_O_7_S, 420.0488; found, 420.0488.

##### 2.2.3.4 2-Amino-5-nitro-N-((4-nitrophenyl)sulfonyl)-1H-benzo [d]imidazole-1-carbox amide (Id)

Yield: 70%; off-white powder; mp: 114–116°C; FTIR (ῡ, cm-1): 3,436 (N–H), 1,643 (C=O), 1,460 (N-O), 1,110 (S=O), ^1^H NMR (DMSO-d6 -250 MHz) δ 9.91 (s, 1H, NH), 7.78 (d, J = 6.70 Hz, 1H, Ar-H), 7.64 (dd, J = 7.48.1.45 Hz, 1H, Ar-H), 7.61 – 7.58 (m, 2H, Ar-H), 7.39 (s, 1H, Ar-H), 7.34 (m, 2H Ar-H), 7.13 (s, 2H, NH_2_). ^13^C NMR (63 MHz, DMSO-d6) δ 167.9 (C=O), 146.9 (C-NH_2_), 146.9 (C-N), 144.3, 144.2, 139.8, 136.1, 128.2, 121.1, 118.6, 116.1, 112.7(Ar-Cs); HRMS (ESI-TOF) m/z: calcd [M+H]^+^ for C_14_H_10_N_6_O_7_S, 406.0331; found, 406.0333.

##### 2.2.3.5 2-Amino-N-((3,5-dichloro-2-hydroxyphenyl)sulfonyl)-1H-benzo [d]imidazole-1-carboxamide (I.e.,)

Yield: 60%; brown solid; mp: 115–117°C; FTIR (ῡ, cm-1): 3,345 (O-H), 2,947 (N–H), 1,660 (C=O), 1,043 (S=O), ^1^H NMR (DMSO-d6–300 MHz) δ 10.10 (s, 1H, NH), 8.86 (s, 1H, OH), 7.84 (d, J = 1.62 Hz, 1H, Ar-H), 7.56 (m, 3H, Ar-H), 7.37 (m, 2H, Ar-H), 7.28 (s, 2H, NH_2_). ^13^C NMR (63 MHz, DMSO-d6) δ 168.7 (C=O), 167.9 (C-OH), 148.4 (C-NH_2_), 132.9 (C-S), 130.2, 128.9, 125.2(C-Cl), 124.0, 122.0, 121.3, 121.0, 118.2, 114.1; HRMS (ESI-TOF) m/z: calcd [M+H]^+^ for C_14_H_10_Cl_2_N_4_O_4_S, 399.9799; found, 399.9796.

##### 2.2.3.6 2-Amino-N-((4-chlorophenyl)sulfonyl)-1H-benzo [d]imidazole-1-carboxamide (If)

Yield: 65%; off-white powder; mp: 110–112°C; FTIR (ῡ, cm-1): 3,429 (N–H), 1,645 (C=O), 1,128(S=O), ^1^H NMR (DMSO-d6 -300 MHz) δ 9.89 (s, 1H, NH), 8.86 (m, 1H, Ar-H), 8.21 (m, 2H, Ar-H), 8.19 (m, 2H, Ar-H), 7.85 (m, Ar-H, 2H), 7.82 (s, 2H, NH_2_), 7.25 (m, 1H, Ar-H).^13^C NMR (75 MHz, DMSO-d6) δ 167.9 (C=O), 154.2(C-NH_2_), 147.2 (C-N), 130.7, 129.1, 127.7, 126.8, 124.0, 121.5, 117.5, 110.8 (Ar-Cs); HRMS (ESI-TOF) m/z: calcd [M+H]^+^ for C_14_H_11_ClN_4_O_3_S, 350.0240; found, 350.0243.

##### 2.2.3.7 2-Amino-N-((2-methyl-5-nitrophenyl)sulfonyl)-1H-benzo [d]imidazole-1-carboxamide (Ig)

Yield: 70%; light yellow solid; mp: 183–184°C; FTIR (ῡ, cm-1): 3,436 (N–H), 1,502 (C=O), 1,048 (S=O), ^1^H NMR (DMSO-d6 - 300 MHz) δ 9.91 (s, 1H, NH), 8.87 (d, J = 1.47 Hz, 1H, Ar-H), 8.51 (dd, J = 7.55, 1.54 Hz, 2H), 8.38 (dt, J = 7.43, 0.95 Hz, 2H), 7.66 (m, 1H, Ar-H) 7.28 (m, 1H, Ar-H), 7.12 (s, 2H), 2.65 (s, 3H, CH_3_).^13^C NMR (126 MHz, DMSO-d6) δ 168.9 (C=O), 168.4 (C-NH_2_), 148.1 (C-NO_2_), 146.9, 145.3, 139.0, 134.4, 132.6, 127.7, 124.7, 123.7, 121.6 (Ar-Cs), 20.9 (CH_3_).); HRMS (ESI-TOF) m/z: calcd [M+H]^+^ for C_15_H_13_N_5_O_5_S, 375.0637; found, 375.0638.

##### 2.2.3.8 2-Amino-5-chloro-N-((3,5-dichloro-2-hydroxyphenyl)sulfonyl)-1H-benzo [d]imidazole-1-carboxamide (Ih)

Yield: 66%; off-white solid; mp: 124–126°C; FTIR (ῡ, cm-1): 3,303 (OH), 2,975 (N–H), 1,650 (C=O), 1,027 (S=O), 887 (C-Cl) ^1^H NMR (DMSO-d6 -300 MHz) δ 9.92 (s, 1H, NH), 8.85 (s, 1H, OH), 8.37 (d, J = 7.01 Hz, 1H, Ar-H), 8.21 (d, J = 3.02 Hz, 2H, Ar-H), 8.04 (d, J = 8.71 Hz, 1H, Ar-H), 7.81 (s, 2H, NH_2_), 7.14 (s, 1H, Ar-H). ^13^C NMR (75 MHz, DMSO-d6) δ 154.1 (C=O), 149.8 (C-NH_2_), 147.2 (C-OH), 144.9, 129.3, 129.1, 129.1, 128.6, 126.9, 126.8, 124.2, 124.0, 117.9, 115.6 (Ar-Cs); HRMS (ESI-TOF) m/z: calcd [M+H]^+^ for C_14_H_9_C_l3_N_4_O_4_S, 433.9410; found, 433.9411.

##### 2.2.3.9 2-Amino-5-chloro-N-((4-chlorophenyl)sulfonyl)-1H-benzo [d]midazole-1-carboxamide Ii)

Yield: 61%; light brown solid; mp: 129–131°C; FTIR (ῡ, cm-1): 3,429 (N–H), 1,502 (C=O), 1,062 (S=O), 889 (C-Cl), ^1^H NMR (DMSO-d6 -250 MHz) δ 9.21 (s, 1H, NH), 7.57 (d, J = 2.4 Hz, 2H, Ar-H), 7.39 (m, J = 8.56 Hz, 4H, Ar-H), 7.35 (d, J = 7.41 Hz, 1H, Ar-H), 7.29 (s, 2H, NH_2_); ^13^C NMR (75 MHz, DMSO-d6) δ 167.9 (C=O), 154.2 (C-NH_2_), 147.2 (C-N), 130.70, 129.1, 127.7, 126.8, 124.0, 121.5, 117.5, 110.8 (Ar-Cs); HRMS (ESI-TOF) m/z: calcd [M + H]^+^ for C_14_H_10_Cl_2_N_4_O_3_S, 383.9850; found, 383.9852.

##### 2.2.3.10 2-Amino-5-chloro-N-((2-methyl-5-nitrophenyl) sulfonyl)-1H-benzo [d]imidazole-1-carboxamide (Ij)

Yield: 72%; off-white solid; mp: 144–146°C; FTIR (ῡ, cm-1): 3,443 (N–H), 1,504 (C=O), 1,032 (S=O), 871 (C-Cl), ^1^H NMR (DMSO-d6- 300 MHz 3) δ 10.79 (s, 1H, NH), 8.06 (d, J = 1.52 Hz, 1H, Ar-H), 7.43 (dd, J = 1.47, 1.46 Hz, 2H, Ar-H), 7.25 (s, 2H, NH_2_), 7.08 (m, 2H, Ar-H), 7.06 (d, J = 7.48 Hz, 1H, Ar-H), 3.12 (s, 3H, CH_3_). ^13^C NMR (126 MHz, DMSO-d6) δ 168.9 (C=O), 168.4 (C-NH_2_), 148.1 (C-NO2), 146.9, 145.3, 139.0, 134.4, 132.6, 127.7, 124.7, 123.7, 121.6 (Ar-Cs), 20.9 (CH_3_); HRMS (ESI-TOF) m/z: calcd [M + H]^+^ for C_15_H_12_ClN_5_O_5_S, 409.0247; found, 409.0248.

##### 2.2.3.11 2-Amino-5-chloro-N-((4-nitrophenyl)sulfonyl)-1H-benzo [d]imidazole-1-carboxamide (Ik)

Yield: 75%; light brown powder; mp: 183–184°C; FTIR (ῡ, cm-1): 3,359 (N–H), 1,502 (C=O), 1,062 (S=O), 881 (C-Cl), ^1^H NMR (DMSO-d6-300 MHz) δ 9.88 (s, 1H, NH), 8.85 (m, 2H, Ar-H), 8.37 (m, 2H), 8.21 (d, J = 1.47 Hz, 1H, Ar-H), 8.18 (d, J = 7.48 Hz, 1H, Ar-H), 8.02 (dd, J = 8.10.1.54 Hz, 1H, Ar-H), 7.85 (s, 2H, NH_2_).^13^C NMR (75 MHz, DMSO-d6) δ 167.9 (C=O), 159.0 (C-NH_2_), 154.2 (C-NO_2_), 147.2, 139.2, 129.6, 129.1, 126.8, 124.0, 123.3 (Ar-Cs); HRMS (ESI-TOF) m/z: calcd [M+H]^+^ for C_14_H_10_ClN_5_O_5_S, 395.0091; found, 395.0091.

##### 2.2.3.12 N-([1.1′-biphenyl]-4-ylsulfonyl)-2-phenyl-1H-benzo [d]imidazole-1-carboxamide (Il)

Yield: 81%; off-white solid; mp: 133–135°C; FTIR (ῡ, cm-1): 3,429 (N–H), 1,502 (C=O), 1,062 (S=O), ^1^H NMR (DMSO-d6-250 MHz) δ 9.42 (s, 1H, NH), 8.10 (m, 2H, Ar-H), 7.81 (m, 2H, Ar-H), 7.69 (m, 3H, Ar-H), 7.60 (m, 3H, Ar-H), 7.41-7.30 (m, 8H, Ar-H). ^13^C NMR (63 MHz, DMSO-d6) δ 150.9 (C=O), 143.3, 142.4, 142.3, 138.6, 130.1, 128.9, 128.8, 126.6, 122.3, 121.3, 119.9, 115.0 (Ar-Cs); HRMS (ESI-TOF) m/z: calculated [M+H]^+^ for C_26_H_19_N_3_O_3_S, 453.1147; found, 453.1148.

##### 2.2.3.13 2-Phenyl-N-(phenylsulfonyl)-1H-benzo [d]imidazole-1-carboxamide (Im)

Yield: 75%; light brown solid; mp: 154–156°C; FTIR (ῡ, cm-1): 3,443 (N–H), 1,627 (C=O), 1,096 (S=O), ^1^H NMR (DMSO-d6-250 MHz) δ 10.29 (s, 1H,NH), 8.32 – 8.30 (m, 2H, Ar-H)), 8.10 (m, 2H, Ar-H), 7.91 (m, 1H, Ar-H), 7.71 (m, 2H, Ar-H), 7.50-7.30 (m, 5H, Ar-H), 7.2 (m, 2H, Ar-H).^13^C NMR (63 MHz, DMSO-d6) δ 150.0 (C=O), 136.1, 131.2, 130.7, 130.1, 129.5, 129.1, 128.2, 127.7, 127.4, 127.1, 123.6, 121.3, 115.0, 114.5 (Ar-Cs); HRMS (ESI-TOF) m/z: calcd [M+H]^+^ for C_20_H_15_N_3_O_3_S, 377.0834; found, 377.0835.

##### 2.2.3.14 N-((4-chlorophenyl)sulfonyl)-2-phenyl-1H-benzo [d]imidazole-1-carboxamide (In)

Yield: 78%; off-white solid; mp: 158–160°C; FTIR (ῡ, cm-1): 3,436 (N–H), 1,641 (C=O), 1,062 (S=O), 706 (C-Cl); ^1^H NMR (DMSO-d6 -250 MHz) δ 9.41 (s, 1H, NH), 8.10-8.00 (m, 2H, Ar-H), 7.90-7.81 (m, 4H, Ar-H), 7.70 – 7.50 (m, 5H, Ar-H), 7.30-7.21 (m, 2H, Ar-H).^13^C NMR (63 MHz, DMSO-d6) δ 150.8 (C=O), 147.1, 142.9, 142.3, 136.4, 132.9, 130.1, 129.5, 129.3, 129.0, 128.9, 127.6, 127.4, 126.6, 122.4, 121.3, 121.2, 115.1, 114.8 (Ar-Cs); HRMS (ESI-TOF) m/z: calcd [M+H]^+^ for C_20_H_14_ClN_3_O_3_S, 411.0444; found, 411.0445.

##### 2.2.3.15 N-((4-nitrophenyl)sulfonyl)-2-phenyl-1H-benzo [d]imidazole-1-carboxamide (Io)

Yield: 80%; white powder; mp: 162–164°C; FTIR (ῡ, cm-1): 3,323 (N–H), 1,688 (C=O), 1,487 (N-O), 1,026 (S=O), ^1^H NMR (300 MHz, DMSO-d6) δ 13.05 (s, 1H, NH), 8.22-8.19 (m, 2H, Ar-H), 7.69 – 7.64 (m, 1H, Ar-H), 7.57 (t, J = 1.8 Hz, 1H, Ar-H), 7.55 (d, J = 1.3 Hz, 2H, Ar-H), 7.53 (d, J = 1.4 Hz, 1H, Ar-H), 7.51 (t, J = 1.5 Hz, 1H, Ar-H), 7.48 (s, 1H, Ar-H), 7.46 (d, J = 2.0 Hz, 1H, Ar-H), 7.21 (tt, J = 9.9, 4.6 Hz, 3H, Ar-H).^13^C NMR (75 MHz, DMSO-d6) δ 161.5 (C=O), 151.1, 150.6, 143.7, 135.0, 130.1, 128.8, 127.6, 127.0, 126.4, 122.4, 121.2, 118.7, 113.9, 112.6, 111.3 (Ar-Cs); HRMS (ESI-TOF) m/z: calcd [M+H]^+^ for C_20_H_14_N_4_O_5_S, 422.0684; found, 422.0685.

##### 2.2.3.16 2-Chloro-N-((4-methoxyphenyl)sulfonyl)-1H-benzo [d]imidazole-1-carboxamide (Ip)

Yield: 80%; off-white solid; mp: 160–162°C; FTIR (ῡ, cm-1): 3,401 (N–H), 1,627 (C=O), 1,026 (S=O), 835 (C-Cl), ^1^H NMR (DMSO-d6-500 MHz) δ 8.22 (s, 1H, NH), 7.90-7.81 (m, 3H, Ar-H), 7.60 (m, 1H, Ar-H), 7.51 (m, 2H, Ar-H), 7.20-7.10 (m, 2H, Ar-H), 3.80 (s, 3H, OCH_3_). ^13^C NMR (126 MHz, DMSO-d6) δ 167.4 (C=O), 159.7, 143.1, 141.8, 136.6, 132.1, 127.5, 120.8, 115.1, 114.4, 113.2 (Ar-Cs), 56.0 (OCH_3_).); HRMS (ESI-TOF) m/z: calcd [M+H]^+^ for C1_5_H_12_ClN_3_O_4_S, 365.0237; found, 365.0238.

##### 2.2.3.17 2-Chloro-N-(phenylsulfonyl)-1H-benzo [d]imidazole-1-carboxamide (Iq)

Yield: 77%; white solid; mp: 179–181°C; FTIR (ῡ, cm-1): 3,457 (N–H), 1,627 (C=O), 1,161 (S=O), 831 (C-Cl), ^1^H NMR (DMSO-d6-300 MHz -) δ 8.10 (s, 1H, NH), 7.80-7.71 (m, 3H, Ar-H), 7.49-7.40 (m, 4H, Ar-H), 7.28-7.25 (m, 2H, Ar-H). ^13^C NMR (75 MHz, DMSO-d6) δ 166.9 (C=O), 142.8, 141.5, 135.4, 131.6, 130.7, 130.1, 128.2, 126.0, 120.2, 114.6 (Ar-Cs); HRMS (ESI-TOF) m/z: calcd [M+H]^+^ for C_14_H_10_ClN_3_O_3_S, 335.0131; found, 335.0133.

##### 2.2.3.18 N-([1.1′-biphenyl]-4-ylsulfonyl)-2-chloro-1H-benzo [d]imidazole-1-carboxamide (Ir)

Yield: 73%; brown solid; mp: 172–174°C; FTIR (ῡ, cm-1): 3,387 (N–H), 1,620 (C=O), 1,161 (S=O), 847 (C-Cl), ^1^H NMR (DMSO-d6-500 MHz) δ 8.10 (1H, NH), 7.93 (m, 1H, Ar-H), 7.88 (m, 3H, Ar-H), 7.86 (m, 2H, Ar-H), 7.74 (m, 2H, Ar-H), 7.73-7.70 (m, 3H, Ar-H), 7.64-7.62 (m, 2H, Ar-H).^13^C NMR (126 MHz, DMSO-d6) δ 167.4 (C=O), 144.1, 143.4, 142.5, 139.1, 132.2, 132.1, 129.4, 128.8, 127.5, 126.7, 126.4, 120.3, 115.1(Ar-Cs); HRMS (ESI-TOF) m/z: calcd [M+H]^+^ for C_20_H_14_ClN_3_O_3_S, 411.0444; found, 411.0443.

##### 2.2.3.19 2-Chloro-N-((4-chlorophenyl)sulfonyl)-1H-benzo [d]imidazole-1-carboxamide (Is)

Yield:84%; light brown solid; mp: 180–182°C; FTIR (ῡ, cm-1): 3,436 (N–H), 1,627 (C=O), 1,160 (S=O), 824 (C-Cl),^1^H NMR (DMSO-d6-500 MHz) δ 8.10 (s, 1H, NH), 7.75 (m, 3H, Ar-H), 7.60 (m, 3H, Ar-H), 7.40 (m, 2H, Ar-H). ^13^C NMR (126 MHz, DMSO-d6) δ 167.4 (C=O), 147.7, 144.5, 142.8, 132.0, 129.5, 128.1, 127.9, 120.1, 115.1 (Ar-Cs); HRMS (ESI-TOF) m/z: calcd [M+H]^+^ for C_14_H_9_Cl_2_N_3_O_3_S, 368.0956; found, 368.0955.

##### 2.2.3.20 2-Chloro-N-((4-nitrophenyl)sulfonyl)-1H-benzo [d]imidazole-1-carboxamide (It)

Yield: 75%; light brown solid; mp: 166–168°C; FTIR (ῡ, cm-1): 3,436 (N–H), 1,627 (C=O), 1,160 (S=O), 842 (C-Cl),^1^H NMR (DMSO-d6-500 MHz) δ 8.20 (s, 1H, NH), 7.72-7.67 (m, 4H, Ar-H), 7.60-7.59 (m, 1H, Ar-H), 7.13 (m, 2H, Ar-H), 6.92 (m, 1H, Ar-H).^13^C NMR (126 MHz, DMSO-d6) δ 167.4 (C=O), 148.7, 145.8, 142.3, 132.1,- 129.1, 128.5, 126.0, 118.0, 115.1 (Ar-Cs); HRMS (ESI-TOF) m/z: calcd [M+H]^+^ for C_14_H_9_ClN_4_O_5_S, 379.9982; found, 379.9984.

##### 2.2.3.21 2-Methoxy-N-((4-methoxyphenyl)sulfonyl)-1H-benzo [d]imidazole-1-carboxamide (Iu)

Yield: 60%; light brown solid; mp: 177–179°C; FTIR (ῡ, cm-1): 3,408 (N–H), 1,627 (C=O), 1,096 (S=O),^1^H NMR (Chloroform-d-500 MHz) δ 8.03 (s, 1H, NH), 7.58 (m, 4H, Ar-H), 7.27 (m, 1H, Ar-H), 7.27 (m, 1H, Ar-H), 7.26 (m, 2H, Ar-H), 3.80 (s, 3H,OCH_3_), 3.76 (s, 3H,OCH_3_).^13^C NMR (126 MHz, DMSO-d6) δ 158.2 (C=O), 156.5, 145.5, 132.0, 128.9, 128.5, 126.1, 123.9, 122.8, 119.9, 109.5, 109.3, 52.4 (O-CH_3_), 50.9 (O-CH_3_).); HRMS (ESI-TOF) m/z: calcd [M+H]^+^ for C_16_H_15_N_3_O_5_S, 361.0732; found, 361.0734.

##### 2.2.3.22 N-((4-chlorophenyl)sulfonyl)-2-methoxy-1H-benzo [d]imidazole-1-carboxamide Iv)

Yield: 65%; light brown solid; mp: 152–154°C; FTIR (ῡ, cm-1): 3,387 (N–H), 1,627 (C=O), 1,160 (S=O), 841 (C-Cl),^1^H NMR (Chloroform-d-500 MHz) δ 7.78 (m, 2H, Ar-H), 7.64 (m, 1H, Ar-H), 7.38 (m, 2H, Ar-H), 7.36 (m, 1H, Ar-H), 7.32 (m, 2H, Ar-H), 1.62 (s, 3H, OCH3). ^13^C NMR (126 MHz, DMSO-d6) δ 158.3 (C=O), 156.2, 145.5, 132.1, 128.2, 128.5, 126.1, 123.9, 122.8, 119.9, 109.5, 109.3, 50.91(O-CH_3_); HRMS (ESI-TOF) m/z: calcd [M+H]^+^ for C_15_H_12_ClN_3_O_4_S, 365.0237; found, 365.0239.

##### 2.2.3.23 2-Amino-N-tosyl-1H-benzo [d]imidazole-1-carboxamide (Iw)

Yield: 70%; pale yellow powder; mp: 165–167°C; FTIR (ῡ, cm-1): 3,390 (N–H), 1,650 (C=O), 1,029 (S=O), 880 (C-Cl),^1^H NMR (DMSO-d6-300 MHz) δ 10.75 (s, 1H, NH), 7.70 (m, 2H, Ar-H), 7.68 (m, 1H, Ar-H), 7.52 (m, 1H, Ar-H), 7.30 (m, 3H, Ar-H), 6.89 (m, 1H, Ar-H), 6.67 (s, 2H, NH_2_) 2.80 (s, 3H, CH_3_). ^13^C NMR (75 MHz, DMSO-d6) δ 169.0 (C=O), 167.9, 159.2, 156.9, 140.8, 139.5, 136.3, 127.0, 125.5, 112.7, 20.3 (CH_3_); HRMS (ESI-TOF) m/z: calcd [M+H]^+^ for C_15_H_14_N_4_O_3_S, 330.0786; found, 330.0785.

##### 2.2.3.24 2-Amino-5-chloro-N-tosyl-1H-benzo [d]imidazole-1-carboxamide Ix)

Yield: 73%; off-white solid; mp: 188–190°C; FTIR (ῡ, cm-1): 3,422 (N–H), 1,502 (C=O), 1,029 (S=O), ^1^H NMR (DMSO-d6-250 MHz) δ 9.91 (s, 1H, NH) 8.87 (m, 2H, Ar-H), 7.75 (d, J = 1.67 Hz, 1H, Ar-H), 7.52 (m, 1H, Ar-H), 7.35 (dd, J = 7.57.1.54 Hz, 1H, Ar-H), 7.15 (m, 2H, Ar-H), 6.95 (s, 2H, NH_2_), 1.85 (s, 3H, CH_3_). ^13^C NMR (63 MHz, DMSO-d6) δ 167.9 (C=O), 138.3, 132.3, 131.8, 130.5, 129.5, 127.7, 127.7, 126.8, 121.5, 113.5, 20.3 (CH_3_); HRMS (ESI-TOF) m/z: calcd [M+H]^+^ for C_15_H_13_ClN_4_O_3_S, 364.0396; found, 364.0394.

##### 2.2.3.25 N-([1.1′-biphenyl]-4-ylsulfonyl)-2-amino-1H-benzo [d]imidazole-1-carboxamide (Iy)

Yield: 70%; off-white solid; mp: 176–178°C; FTIR (ῡ, cm-1): 3,380 (N–H), 1,627 (C=O), 1,128 (S=O), ^1^H NMR (DMSO-d6-500 MHz) δ 8.02 (1H, NH), 7.88 (q, J = 8.3 Hz, 1H, Ar-H), 7.77 – 7.71 (m, 1H, Ar-H), 7.71 – 7.63 (m, 1H, Ar-H), 7.57 – 7.45 (m, 1H, Ar-H), 7.39 – 7.25 (m, 1H, Ar-H), 7.19 (d, J = 7.0 Hz, 1H, Ar-H), 7.12 – 7.05 (m, 4H, Ar-H), 6.95 (td, J = 7.6, 1.2 Hz, 1H), 6.84 (dd, J = 5.6, 3.1 Hz, 5H). ^13^C NMR (126 MHz, DMSO-d6) δ 155.9, 155.8, 155.7, 154.1, 152.4, 149.0, 139.5, 137.3, 134.4, 131.2, 129.5, 128.2, 121.2, 120.0, 119.4, 114.6, 101.9. HRMS (ESI-TOF) m/z: calcd [M+H]^+^ for C_20_H_16_N_4_O_3_S, 392.0943; found, 392.0944.

### 2.3 Biological activities

#### 2.3.1 Cell culture

Dr. Gary A. Weisman provided human-1321N1 astrocytoma cells that had been transfected by the tP2Y1R, hP2Y2R, hP2Y4R, and rP2Y6R and untransfected 1321N1 astrocytoma cells (University of Missouri Mo., Bond Life Science Center). The DMEM Dulbecco’s Modified Eagle -Medium (Gibco) was added in flasks to cultivate the cell line. 10% of the Fetal bovine serum was also added to the media together with 500 μg/mL of Geneticin (G418) and 1% of the Pen/Strep. The cells in T-75 flask were then cultured and incubated in a CO_2_ incubator which was maintained at (5%) and 37°C temperature until 90% of the cells were confluent. Throughout the experiment, cells were periodically checked for the confluency and strictly watched for the microbial contamination.

#### 2.3.2 MTT assay

A 96-well plate was used to plate 20,000 human 1321N1 astrocytoma cells and allowed for incubation for 24 h at temp of 37°C in CO_2_ (5%) incubator. Next to this, the prepared culture media was changed to media which was free from FBS, the test compounds were added and the incubation process was repeated. After incubation of 24 h, the serum free media from the wells was discarded, and 100 μL of the reagent i.e., MTT reagent was pipetted. Plate was then additionally incubated at 37°C for 4 h. Utilizing a microplate reader, absorbance was determined at 570 nm and 630 nm for calculation of cell viability (FLUOstarOmega Microplate Reader-BMG LABTECH GmbH, Ortenberg, Germany) (Sauer et al., 2009).

#### 2.3.3 Analysis of t-P2Y1, h-P2Y2, h-P2Y4, and r-P2Y6 receptors expression in h- 1321N1 astrocytoma cell line

Real-time PCR was used to assess the efficacies of targeted genes (t-P2Y1, h-P2Y2, h-P2Y4, and r-P2Y6). For this purpose, extraction of RNA was done by using reagent Trizol which was bought from (Invitrogen-Thermo Fisher Scientific) from stable transfected cell lines. According to the manufacturer instructions (Thermo Scientific), 2 μg mRNA of each desired gene (quantified by LVis plate) was occupied for the synthesis of cDNA using Reverse Transcription Kit. Program settings for the thermocycler were 25°C for time period of 5 min, 42°C for time period of 70 min, 70°C temp for 5 min, and infinity at 8°C. For RT-PCR, a master mixture which contains 1.5 μL = cDNA, 0.25 μL = reverse primer, 0.25 μL = forward primer, 10 μL = SYBR Green, and 8 μL from RNAse-free water was utilised, yielding a master volume of around 20 μL for each sample (Primer sequences are listed in Table 1). The following settings and programming were utilised in the next step with the PikoReal RT- PCR System such as, 95°C for 2 min in the first step, followed by second step of forty cycles with temp of 95°C for time of 30 s, 60°C; 30 s, and final extension step for 1 min at temp of 72°C. After the run was completed, the products from PCR were allowed to run on agarose gel (2%), and gel images were taken by Imaging System (ProteinSimple AlphaImager Mini Imaging system) (Mahmood et al., 2021).

**TABLE 1:** Primer Sequence of *t-*P2Y1, *h*-P2Y2, *h*-P2Y4, and *r*-P2Y6 receptors for RT-PCR.

Primer name	Sequence (5′–3′)
*t*-P2Y1R (F)	TAC​ATG​TTC​AAC​CTG​GCG​CT
*t*-P2Y1R (R)	CAT​CCC​CGA​AGA​TCC​AGT​CG
*h*-P2Y2R (F)	TCC​TGT​TTC​CCG​CAG​AGT​TC
*h*-P2Y2R (R)	CAC​CTG​ACC​AGG​GCT​TTC​AT
*h*-P2Y4R (F)	CCT​GGC​ATT​GTC​AGA​CAC​CT
*h*-P2Y4R (R)	GAA​AGC​GGA​CGA​ACT​TGC​AG
*r*-P2Y6R (F)	TAG​GTG​AAA​GCA​GGC​AAC​GA
*r*-P2Y6R (R)	TCC​CTC​TCA​GCC​TCA​AGC​TA

#### 2.3.4 Calcium mobilization assay

The FLUOstar Galaxy (BMG LABTECH GmbH, Ortenberg, Germany) microplate reader with two injectors was used to measure the Ca^2+^ fluorescence of synthetic compounds. For this assay, 1321N1 transfected cells (t-P2Y1, h-P2Y2, h-P2Y4, and r-P2Y6) were cultured in T-75 flask. When cells reached the confluency of 80%–90%, they were washed with PBS and then Trypsin was added. After trypsinization for 3 min, 20,000 cells/well were allowed to seed in a black wall and 96 well clear bottom plate which was coated with Poly-D-Lysine and allowed to incubate for 24 h in 37°C, 5% CO_2_ incubator. After incubation, 200 μL culture media was changed with 100 μL HBSS containing Fura-2 AM which is calcium chelating dye and allowed the plate to incubate for 45 min in dark at temp of 37°C in incubator which is maintained with carbon dioxide (5% CO_2_). After incubation for 45 min, synthesized molecules were added to HBSS washed cells in each well of 96 well plate and re-incubated for 30 min at 37°C. In next step, injection of the endogenous ligand such as ADP through the injector was followed by excitation and emission measurements of Fura-2 AM at 380/520 nm and 340/520. Final volume of the assay per well 200 μL. IC_50_ values were computed for the compounds displaying more than 50% of activity using Prism 5.0 software (Graph Pad., Inc., San Diego. California, United States) by using analysis i.e., nonlinear regression (Kassack et al., 2002; Ullmann et al., 2005).

#### 2.3.5 Aqueous solubility assay

In eppendorf tubes, over-saturated chemical solutions were prepared by mixing synthesized compound with PBS buffer (pH 7.4). To confirm compound saturation, suspensions were rapidly vortexed for 1 min, sonicated at ambient temperature for 10 min, and then incubated with overnight shaking. After that, compound suspensions were centrifuged at 15,000 RPM for 20 min. Supernatants were cautiously taken out of the tubes and filtered once more using 0.22 m syringe filters. By using UV-vis absorption spectrometry with standard curves and an already-determined absorption extinction coefficient in the same buffer, the concentrations of the drug in the supernatants were identified.

#### 2.3.6 Docking studies

For understanding the binding mechanisms and validate the *in vitro* results of the sulfonylurea derivatives of benzimidazole (**1a-y**) towards P2Y receptor, docking was conducted. Crystal structure of P2Y1R with PDB ID = 4XNV was used for this study (Le et al., 2019). PDB ID was downloaded from RSCB (https://www.rcsb.org/). Targeted proteins were prepared by using software (MOE, 2019) with default parameters, the energy minimization and amino acid residues sequence correction was done through quick preparation, after that active amino acid residues were selected, and then docking was performed by applying dummies atoms. The ligand structure were sketched by using Chemdraw 15.0 and the energy minimization were also done in 3D Chem draw after selection of prepared ligand then run docking of top 20 poses. Using Discovery Studio Visualizer DS, the best poses with low binding affinity and highest binding affinity were chosen and examined (Discovery Studio Visualizer DS, 2017).

## 3 Results

### 3.1 Chemistry

**FIGURE 2:**
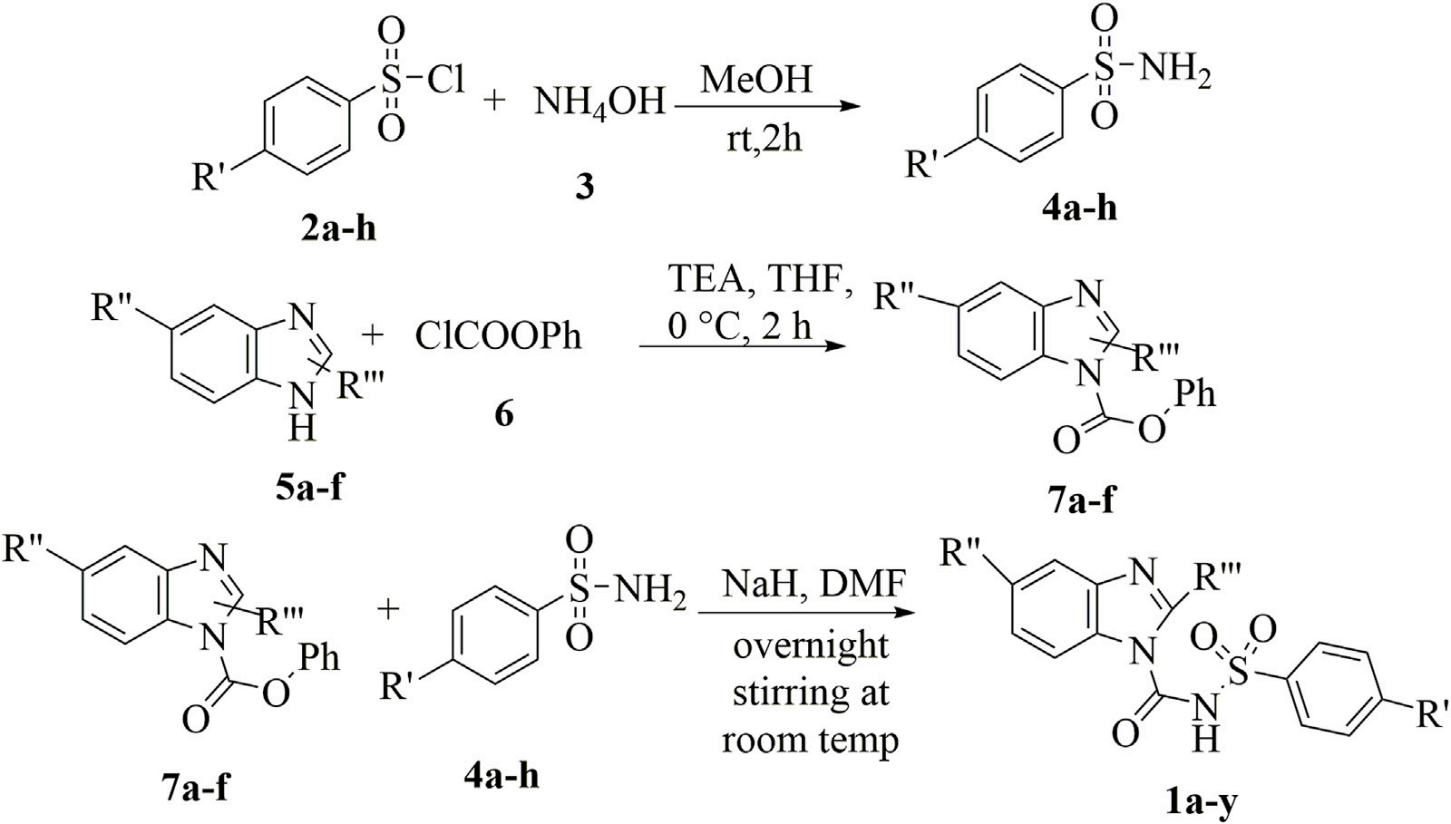
Schematic diagram for synthesis of benzimidazole based sulfonylurea derivatives.

Sulfonylurea analogues were synthesized in a three steps of reaction as depicted in Figure 2 and all substitutions are coded in Table 2. In first step, synthesis of appropriate aryl sulfonamides by reacting suitable arylsulfonyl chlorides with (NH_4_OH) ammonium hydroxide was done. In second step, interaction of benzimidazole and phenyl chloroformate with triethylamine (TEA) as catalyst produced the intermediates of carbamate. The twenty-five target sulfonylurea products in good to excellent yield were synthesized in last and final step by reacting the synthesized sulfonamides (**4a-h**) and carbamates (**7a-f**) for 15h with sodium hydride as catalyst.

**TABLE 2:** Synthesized analogues (1a-y).

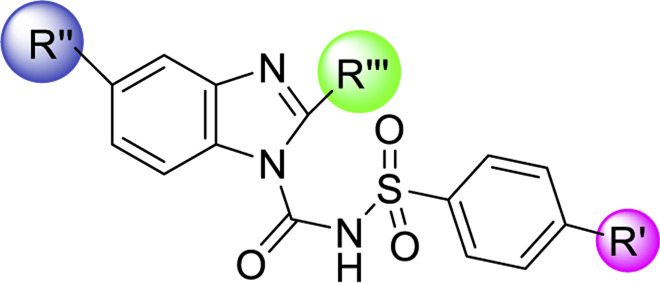							
Code	R'	R″	R‴	Code	R'	R″	R‴
**1a**	3.5- *di*Cl, 2-OH	NO_2_	NH_2_	**1k**	4-NO_2_	Cl	NH_2_
**1b**	4-CH_3_	NO_2_	NH_2_	**1l**	4-C_6_H_5_	H	C_6_H_5_
**1c**	2-CH_3_, 5-NO_2_	NO_2_	NH_2_	**1m**	H	H	C_6_H_5_
**1d**	3-CH_3_	NO_2_	NH_2_	**1n**	4-Cl	H	C_6_H_5_
**1e**	3.5- *di*Cl, 2-OH	H	NH_2_	**1o**	4- NO_2_	H	C_6_H_5+_
**1f**	4-Cl	H	NH_2_	**1p**	4-OCH_3_	H	Cl
**1g**	2-CH_3_, 5-NO_2_	H	NH_2_	**1q**	H	H	Cl
**1h**	3.5- *di*Cl, 2-OH	Cl	NH_2_	**1r**	4-C_6_H_5_	H	Cl
**1i**	4-Cl	Cl	NH_2_	**1s**	4-Cl	H	Cl
**1j**	2-CH_3_, 4-NO_2_	Cl	NH_2_	**1t**	4-NO_2_	H	Cl
**1u**	4-OCH_3_	H	OCH_3_	**1v**	4-Cl	H	OCH_3_
**1w**	4-CH_3_	H	NH_2_	**1x**	4-CH_3_	Cl	NH_2_
**1y**	4-C_6_H_5_	H	NH_2_				

Bold text is the code of the synthesized compounds

Physical properties of synthesized compounds (**1a-y**) such as colour and melting points were determined. Structure elucidation of compounds was done through FTIR, ^13^C NMR, ^1^H NMR and HRMS. In spectra of FTIR, absorbance bands for different functional groups appeared at appropriate ranges confirmed the synthesis of various analogues. Stretching frequency around 3,300 and 3,400 cm-1 relate to N-H while the stretching of carbonyl (C=O) appeared around 1,680 and 1,500 cm^-1^ confirmed the functionalities. In case of ^1^H NMR, the characteristic broad signals between 7.30ppm and 10.81ppm were observed for N-H respectively. Peaks for the protons of benzimidazole ring were detected in a range of 6.65–8.80 ppm. In ^13^C-NMR, all of the synthesized molecules display characteristic signals at δ 168.78, 167.92 for carbonyl carbon.

### 3.2 Biological assays

#### 3.2.1 Compound toxicity and cell viability study

Cell viability assay was performed to evaluate the cytotoxic nature of the effective compounds and lower the possible effects of calcium influx that might be produced by cell toxicity producing false positive results. The cytotoxic potential of synthesized sulfonylureas was evaluated by using the (MTT) [3-(4, 5-dimethylthiazol-2-yl)-2, 5- diphenyl-tetrazolium bromide] assay. Among the anti-cancer drugs cisplatin is used as standard for positive control. The results are shown in Table 3. None of the sulfonylurea derivatives were identified as cell toxic, when the synthesized molecules were investigated with dose up to 50 μM. As a result, it was believed that any inhibitory impact seen by calcium influx assays was unlikely to be caused by the cytotoxicity of synthesized series (1a-y).

**TABLE 3:** Cytotoxic potential of compounds **(1a-y)** (% inhibition ±SEM) towards HEK-293**,** non-transfected astrocytoma 1321N1 cells, and HeLa cell line at 50 µM.

Compound	H-1321N1 Cell Line (%cytotoxicity ±SEM)^a^	HEK-293 Cell Line (%cytotoxicity ±SEM)^a^	HeLa Cell Line (%cytotoxicity ±SEM)^a^
**1a**	27.4 ± 0.5	13.3 ± 0.1	22.2 ± 1.1
**1b**	17.3 ± 0.8	21.3 ± 0.2	21.6 ± 0.2
**1c**	21.1 ± 0.6	19.5 ± 0.4	26.4 ± 0.7
**1d**	15.9 ± 0.3	20.3 ± 0.6	28.3 ± 0.4
**1e**	39.9 ± 0.2	16.3 ± 0.2	21.3 ± 0.6
**1f**	23.6 ± 0.8	21.2 ± 0.7	16.4 ± 0.2
**1g**	46.2 ± 0.2	14.3 ± 0.3	11.6 ± 1.1
**1h**	32.8 ± 0.7	27.4 ± 1.2	22.3 ± 0.8
**1i**	38.7 ± 0.8	36.2 ± 0.1	39.3 ± 0.4
**1j**	44.3 ± 0.6	38.2 ± 0.4	26.1 ± 0.7
**1k**	41.7 ± 0.2	27.2 ± 0.2	21.7 ± 0.9
**1l**	47.2 ± 0.4	21.3 ± 0.6	28.3 ± 1.2
**1m**	37.9 ± 0.7	33.3 ± 0.3	39.4 ± 1.3
**1n**	26.5 ± 0.4	38.7 ± 0.3	22.4 ± 0.8
**1o**	24.3 ± 0.8	27.9 ± 0.8	24.3 ± 0.4
**1p**	21.2 ± 0.3	24.2 ± 0.7	20.5 ± 0.2
**1q**	33.6 ± 0.7	30.1 ± 0.2	29.7 ± 1.1
**1r**	31.5 ± 0.4	33.2 ± 0.1	26.5 ± 0.5
**1s**	14.4 ± 0.2	12.1 ± 0.7	19.5 ± 0.2
**1t**	27.6 ± 0.4	21.4 ± 1.1	20.3 ± 0.4
**1u**	25.5 ± 0.3	32.2 ± 0.9	21.7 ± 0.6
**1v**	19.7 ± 0.1	12.2 ± 0.7	24.1 ± 0.3
**1w**	23.2 ± 0.3	27.4 ± 0.3	21.3 ± 0.5
**1x**	12.6 ± 0.5	32.1 ± 0.7	29.8 ± 1.5
**1y**	43.1 ± 0.8	39.7 ± 0.2	32.6 ± 0.3
**Suramin**	12.3 ± 0.2	11.1 ± 0.2	37.5 ± 0.2
**Cisplatin**	85.4 ± 0.2	90.4 ± 0.9	86.3 ± 0.6

^**a**^ Experiments performed in triplicate format.

#### 3.2.2 Expression study of P2YRs in h-1321N1 astrocytoma cell line

**FIGURE 3:**
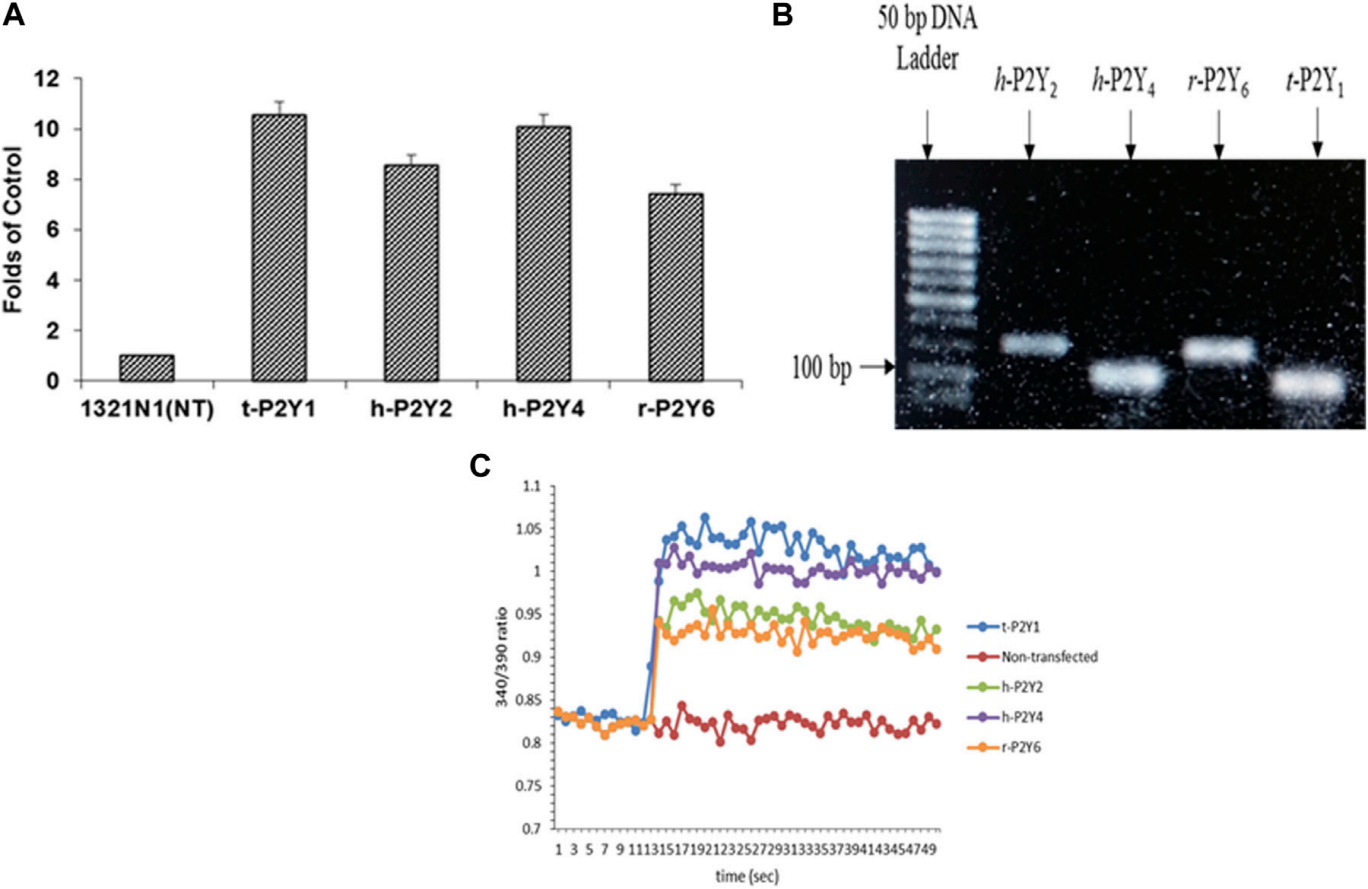
Expression analysis of t-P2Y1, h-P2Y2, h-P2Y4, and r-P2Y6Rs expressed in astrocytoma h-1321N1 cells through RT-PCR and then RT-PCR products run on gel electrophoresis **(A, B)**. Stimulated non-transfected, t-P2Y1, h-P2Y2, h-P2Y4, and r-P2Y6 receptors with 100 μM ADP for P2Y1R, 500 μM ATP for P2Y2R, 500 μM UTP for P2Y4R and 100 μM of UDP for P2Y6R **(C)**.

For detection and confirmation of expressions efficiency of P2YRs transfected in astrocytoma 1321N1 cells, reverse transcription polymerase chain reaction was used plus non-transfected h-1321N1 astrocytoma cells were employed as control. In comparison to the control, it was discovered that r-P2Y6R was eight times as expressed as t-P2Y1R, h-P2Y2R was nine times as expressed, and hP2Y4R was ten times as expressed (Figure 3A). Strong bands between 100 and 200 bp were seen on a 2% agarose gel after RT-PCR products were run. (Figure 3B). The amplicon size of product retained when constructing the h-P2YR primers is confirmed by the existence of product of RT-PCR in this range of base pair. Ca^2+^ flow functional assay was used to further establish P2YR expression. Here, Ca^2+^ influx through these gates was activated by using ADP (500 nM) for t-P2Y1, ATP (500 nM) for h-P2Y2, UTP (500 nM) for h-P2Y4, and UDP (800 nM) in the case of r-P2Y6 (Figure 3C). It was shown that the injection of an agonist into P2Y receptor-expressed cell lines caused a sudden increase in fluorescence signals. When compared to a cell line that had not been transfected, this functional experiment demonstrated the expression of receptor proteins.

#### 3.2.3 Calcium mobilization assay and structure-activity relationship studies (SAR)

All the synthesized compounds were evaluated for inhibition rate and IC_50_ test against four targeted receptors with the specific aim to identify these derivatives as potential antagonist of P2Y1 receptor. The antagonistic activity was measured by a calcium mobilization assay in which suramin and reactive blue were used as positive controls. This characteristic assay was performed on stably transfected h-1321N1 astrocytoma cell lines which were transfected with P2Y1, Y2, Y4 and Y6Rs. The activity was also compared with the selective P2Y1 antagonist i.e., 1-(2-(2-tert-butylphenoxy)pyridin-3-yl)-3-4-(trifluoromethoxy) phenylurea having IC_50_ value of 0.28 ± 0.24 µM, reported earlier. The results are reported as inhibition rate at (100 μM and the inhibitory concentration values for only those compounds exhibited 50% inhibition (IC_50_). The structure activity relationship for these compounds is summarized in Table 4.

**TABLE 4:** Potencies of synthesized derivatives measured through Ca^2+^ influx inhibitory assay.

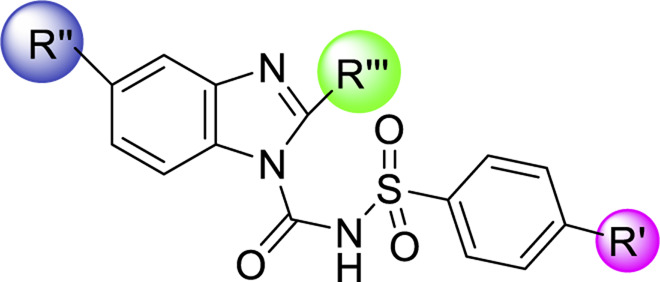							
Code	R’	R″	R‴	IC_50_ ± SEM (µM)^*a*^/% Inhibition			
				*t*-P2Y1	*h*-P2Y2	*h*-P2Y4	*r -*P2Y6
**1a**	3.5- *di*Cl, 2OH	NO_2_	NH_2_	0.37 ± 0.02	44%	41%	0.66 ± 0.04
**1b**	4-CH_3_	NO_2_	NH_2_	33%	38%	18%	21%
**1c**	2-CH_3_, 5NO_2_	NO_2_	NH_2_	11.11 ± 0.04	6.32 ± 1.10	42%	16.05 ± 0.59
**1d**	3-CH_3_	NO_2_	NH_2_	43%	41%	48%	39%
**1e**	3.5- *di*Cl, 2OH	H	NH_2_	0.46 ± 0.01	5.50 ± 1.21	0.60 ± 0.01	0.75 ± 0.08
**1f**	4-Cl	H	NH_2_	0.94 ± 0.01	8.41 ± 0.07	47%	9.30 ± 1.12
**1g**	2-CH_3_, 5NO_2_	H	NH_2_	11.18 ± 0.71	33%	13%	40%
**1h**	3,5*di*Cl, 2OH	Cl	NH_2_	0.19 ± 0.04	4.13 ± 1.02	0.91 ± 0.04	34%
**1i**	4-Cl	Cl	NH_2_	1.21 ± 0.07	15.66 ± 1.48	34%	41%
**1j**	2-CH_3_,4NO_2_	Cl	NH_2_	13.44 ± 0.22	32%	42%	25.37 ± 3.10
**1k**	4-NO_2_	Cl	NH_2_	12.42 ± 1.03	11.11 ± 0.94	16.64 ± 1.12	8.49 ± 1.23
**1l**	4-C_6_H_5_	H	C_6_H_5_	27%	34%	22%	43%
**1m**	H	H	C_6_H_5_	29%	37%	16%	21%
**1n**	4-Cl	H	C_6_H_5_	5.69 ± 0.23	21%	19%	7.06 ± 1.47
**1o**	4- NO_2_	H	C_6_H_5_	46%	18.18 ± 0.77	46%	32%
**1p**	4-OCH_3_	H	Cl	5.59 ± 0.91	12%	20%	16.30 ± 0.83
**1q**	H	H	Cl	14.65 ± 1.01	13.62 ± 1.97	13%	48%
**1r**	4-C_6_H_5_	H	Cl	23.44 ± 0.22	47%	22%	34%
**1s**	4-Cl	H	Cl	3.11 ± 0.23	6.15 ± 1.08	2.67 ± 0.07	12.68 ± 1.02
**1t**	4-NO_2_	H	Cl	6.04 ± 1.03	42%	26%	12.36 ± 1.10
**1u**	4-OCH_3_	H	OCH_3_	31%	10%	30%	18%
**1v**	4-Cl	H	OCH_3_	42%	19%	23%	19%
**1w**	4-CH_3_	H	NH_2_	49%	23%	41%	39%
**1x**	4-CH_3_	Cl	NH_2_	10.04 ± 0.12	38%	29%	41%
**1y**	4-C_6_H_5_	H	NH_2_	12%	20%	18%	23%
**Suramin**				0.15 ± 0.01	3.31 ± 0.08	n.d	n.d
**Reactive Blue 2**				n.d	0.57 ± 0.02	8.4 ± 1.2	2.8 ± 0.11

^α^ Calcium mobilization inhibition in astrocytoma 1321N1 cells transfected with human P2YRs, followed by activation of receptor with 100 μM ADP (P2Y1R), 500 μM ATP (P2Y2r), 500 μM UTP (P2Y4R) and 100 μM of UDP (P2Y6R). Percent inhibition was calculated at final conc of 100 μM. (n.d = not determined). (t = turkey, h = human, r = rat).

**FIGURE 4:**
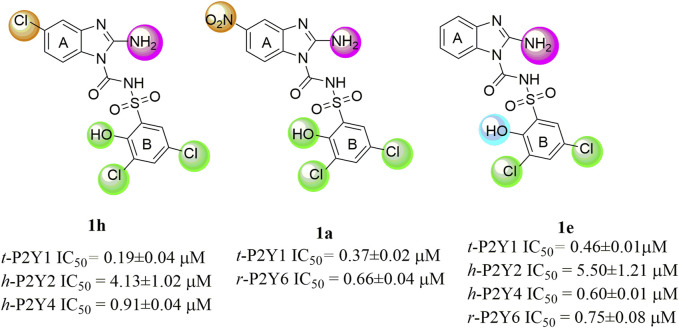
Structures and IC_50_ values of the most potent compounds.

**FIGURE 5:**
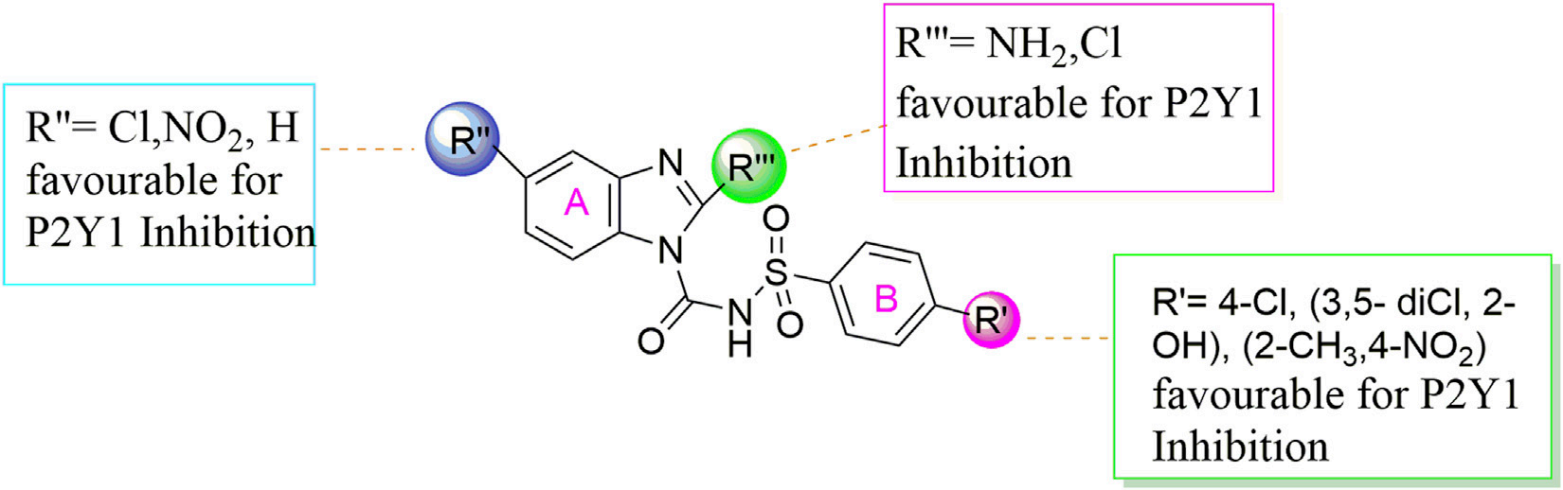
Summary of SAR of the target compounds against P2Y1 receptor.

**FIGURE 6:**
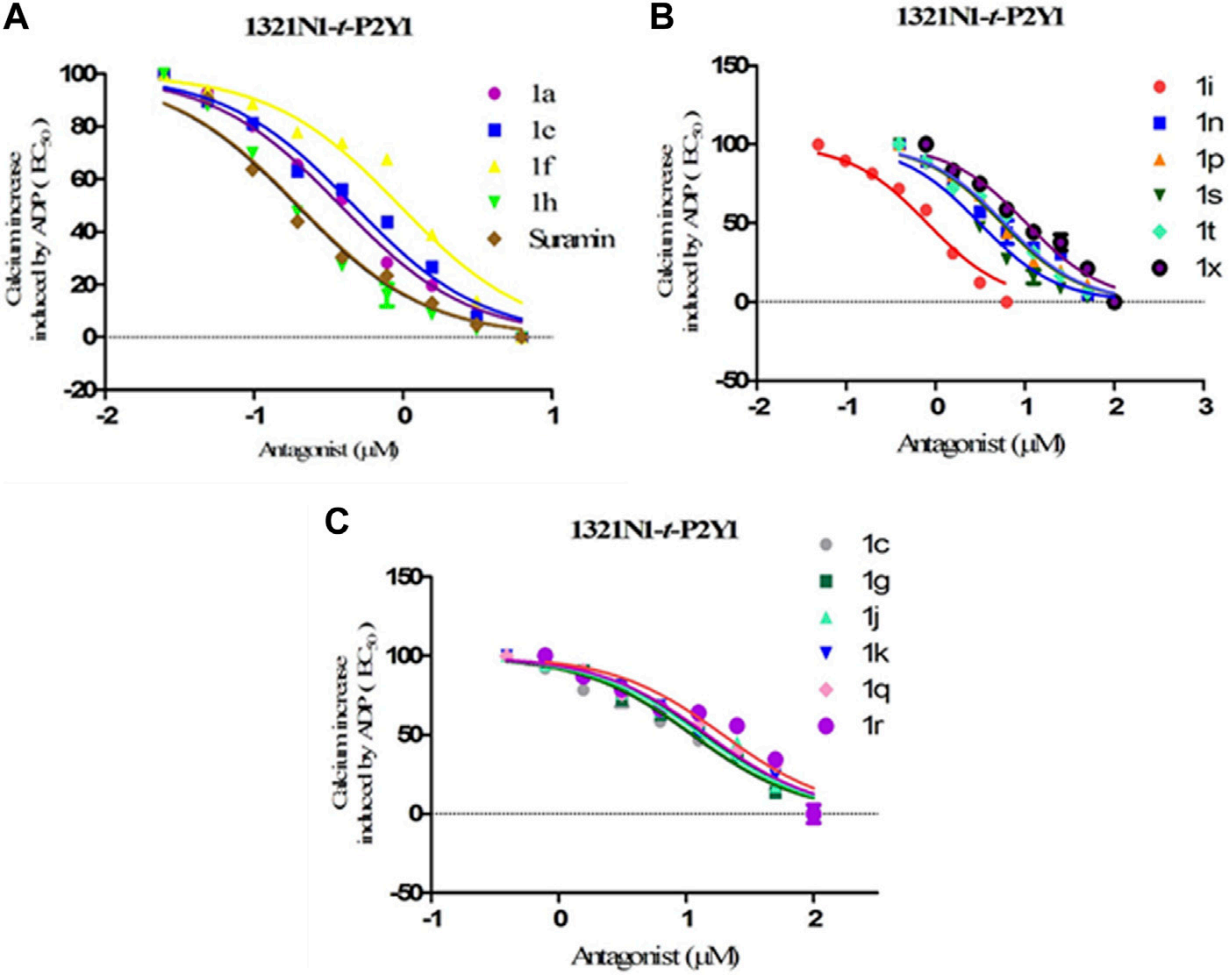
Dose-response curves for transfected *t* **-**P2Y1-1321N1 cells; ADP (100 μM) was added as an agonist for screening compounds 1a-y against P2Y1 receptor. **(A)** Inhibition curves for compounds 1a, 1e, 1f, 1h and Suramin **(B)** Inhibition curves compounds 1i, 1n, 1p, 1s, 1t and 1x **(C)** Inhibition curves for compounds 1c,1g,1j,1k, 1q, and 1r.

The structure activity relationship was studied based on a variety of structural modification among different derivatives which contain benzimidazole nucleus and sulphonyl urea linkage. Among all the synthesized derivatives, compound **1b, 1d, 1l, 1m, 1o, 1u, 1v, 1w** and **1y** depicted less than 50% inhibition of all the targeted receptors while derivative **1a, 1e,** and **1h** were found potent derivatives exhibited strong inhibition of P2Y1 receptor. All these three derivatives have same R′ and R‴ but different R″group. Compound **1h** exhibited more potential as compared to **1a** and **1e** with an IC_50_ values of 0.19 ± 0.04, 0.37 ± 0.02 and 0.34 ± 0.01 µM (Figure 4). The detailed structure activity relationship suggested that the presence of electronegative functional group is important for the inhibition of P2Y1 receptor as it can be observed in case of **1h**, where R″ the Cl functional group was introduced at position 4. Among all the tested derivatives this **1h** was identified the most potent and selective inhibitor of P2Y1 receptor. This derivative also exhibited inhibition of P2Y2 but 20 fold less as compared to P2Y1. To understand the effect of substitutional functional groups, the compounds are divided into different sets i.e., having same R″ and R‴substitution. The compounds i.e., **1a, 1b, 1c** and **1d** have NO_2_ and NH_2_ as R″ and R‴ but different R′ group i.e., and 3,5- diCl, 2-OH, 4-CH_3_, H and 3-CH_3_ functional groups respectively. The methyl substitution was found unfavorable for the receptor inhibition irrespective of the position However, in case of no substitution the compound exhibited inhibition of P2Y1 and P2Y2 selectively (Figure 5). The activity was improved when electronegative substitution was introduced i.e., in **1a**. When the structure of **1b** was compared with **1w** and **1x** it was observed that derivative **1w** also found inactive against all the targeted receptors however, derivative **1x** having Cl functional group at R″ position showed inhibition of P2Y1 selectively. Interestingly, this behavior was observed in case of all compounds having Cl group substitution. However, both 3 and 5 positions were found more favorable in comparison to 4 position substitution. The compound **1f** with chloro (Cl) at *para* position on ring B and no substitution at *meta* position on ring B resulted in the decreased potency of about five times relatively to the derivative **1h**. The inhibition curve of these potent inhibitor showed in Figure 6. The replacement of R‴ with methoxy group resulted in the loss of activity as can be observed in case of derivative **1u** and **1v**. The compound **1v** having 4-Cl group did not show any effect but better %inhibition result as compared to **1u**, only because of the presence of electronegative substitution. Similarly when R‴ is replaced by Cl group like in **1p, 1q, 1r, 1s** and **1t** resulted in the different response. Among these derivatives the compounds, the derivative **1s** having R′ as 4-Cl substitution exhibited excellent inhibition of all the selected receptors non-selectively but found more potent towards P2Y1. Here, the substitution with the methoxy group resulted in the loss of activity towards all the targeted receptors except P2Y1 just because of presence of Cl irrespective of the position. An interesting behaviour was observed when the structure activity relationship of those derivatives was studied having phenyl ring at position R‴ i.e., derivative **1l, 1m, 1n** and **1o**, it was observed that only **1n** exhibited some inhibitory potential towards P2Y1 and P2Y6 but did not show any response towards P2Y2 and P2Y4 receptor. This derivative showed inhibition only because of the presence of 4-Cl substitution (at R′).

Conclusively, the SAR study of these compounds presented that existence of groups which are electron withdrawing i.e., chloro (Cl) substituent at m-position on benzene sulfonylurea moiety reserved the activity towards P2Y1 receptor suggested that chloro substitution was favorable at benzene sulphonyl moiety. While compounds with an alkyl moiety (-CH_3_) i.e., compounds **1b** and **1d** at benzene sulphonyl moiety showed no antagonistic effect against P2Y1, 2, 4 and P2Y6Rs. But the attachment of nitro group alone and together with alkyl group at benzene sulphonyl moiety (compound **1k, 1t, 1g and 1j**) showed inhibition against P2Y1 receptor with IC_50_ value of 12.42 μM, 6.04 μM, 11.18 μM and 13.44 μM respectively which revealed that nitro group is favourable for antagonistic activity against P2Y1 receptor. The presence of bulky groups such as (-C_6_H_5_ and -OCH_3_) showed no favourable activity. Therefore, it can be suggested that introduction of electronegative Cl group resulted in the improved inhibition of the targeted receptors however, the position 3 and position 5 resulted in the improved inhibition of P2Y1 receptor.

*In silico* studies of the most potent compounds were also performed which support *in vitro* results. Compound 1 h showed considerable interaction comparable with BPTU at the allosteric site located on the outer hydrophobic surface of the transmembrane (TM) bundles and is in contact with the phospholipid bilayer of the receptor. BPTU is a non-nucleotide antagonist of P2Y1R belonging to aryl urea derivatives while our synthesized compounds belong to the same family showing similar characteristic and behaviour. In case of compound 1h, NH of sulphonyl urea linkage, Oxygen of SO_2_ and free NH_2_ group of the benzimidazole ring show H-bonding with Leu102, Ala106 and Gln127 respectively. Chloro (-Cl) at meta position of benzimidazole ring show π-alkyl interaction, with Leu102 and chloro (-Cl) groups attached at meta position of phenyl ring showed π-alkyl interaction with Phe119 and Met123 respectively. Similarly, compound **1a, 1e** and **1f** showed same binding mode of interaction as compare with the **1h** due to structural similarity among them. Some extra H-bonding interaction were observed in our synthesized compounds such as Gln127and Ala106 which are not present in the standard antagonist.

#### 3.2.4 Solubility assay

**FIGURE 7:**
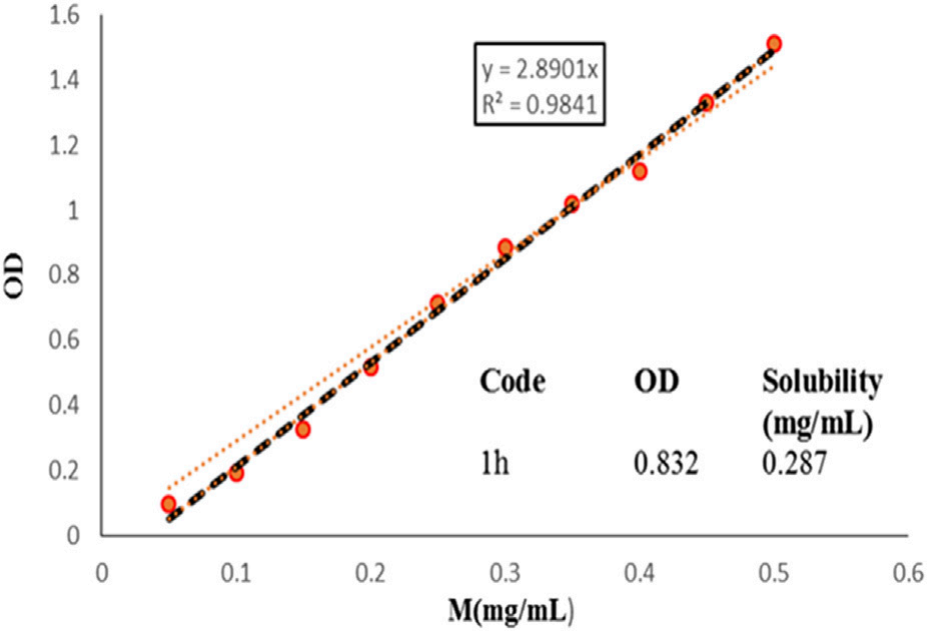
Aqueous solubility of 1h, measured by UV absorption spectroscopy. The optical density values were recorded at 384 nm.

An important aim of synthesized compounds was to increase the aqueous solubility of compounds. For this purpose, solubility assay was performed for the most potent compound (**1h**) of series (**1a-y**) in aqueous solution using UV absorption spectroscopy, respectively (Figure 7). BPTU has an extremely low solubility in water, less than 0.1 mg/mL, was reported. The aqueous solubility of **1h**, however, has significantly increased and stands at 287 μg/mL, respectively. The improved aqueous solubility of compound **1h** provides a way to evaluate their functional profile *in vivo*.

#### 3.2.5 In silico ADME studies

Chemdraw 15.0 was used to generate the SMILES of chemical structures for the evaluation of drug-likeness factors, and friendliness as a medicinal chemistry item. The SMILES database was added to the Swiss ADME online service. The Swiss ADME online service is a web-based tool that uses computational models to predict ADME properties of compounds based on their molecular structure. Supplementary Table S3 provides an overview of the collected data, which includes the predicted ADME properties, and their compliance with Lipinski’s rule of five. Most of the compounds evaluated in this study showed good gastrointestinal absorption and were compliant with the Lipinski’s rule of five, possessing no more than three hydrogen bond donors and eight hydrogen bond acceptors. Overall, the use of Chemdraw to generate SMILES notation, and the subsequent analysis of ADME properties and drug-likeness factors, provides valuable insights into the potential of the compounds to be developed as drugs, which can inform future drug discovery and development efforts.

#### 3.2.6 Docking studies

*In silico* studies were conducted to investigate the manner in which compounds binds with the active site of P2Y1 receptor. P2Y1R crystal structure: **PDB ID = 4XNV** was downloaded to identify the potential binding interactions with the synthesized antagonists. Among all of compounds, compounds **1a, 1e,** and **1h** showed the excellent inhibition towards P2Y1R in functional assay. In order to further evaluate them, molecular docking investigations were conducted. In docking studies compound **1a, 1e,** and **1h** show high binding energies towards the P2Y1 receptor i.e., −6.19, −5.59, and −6.02 kcal/mol respectively.

BPTU, the reported antagonist of P2Y1 receptor was re-docked in allosteric binding pocket of P2Y1R with binding energy of −7.04 kcal/mol. Leu102 showed the most promising H-bonding interaction with the nitrogen atom of the urea linkage. Pro105, Leu126, Ala106 showed pi-alkyl interactions while, Thr103, Met123, Phe119 and Phe62 residues showed pi-sigma interactions (Zhang et al., 2015). These mentioned hydrophilic and hydrophobic interactions were considerable for the inhibitory activity of BPTU.

**FIGURE 8:**
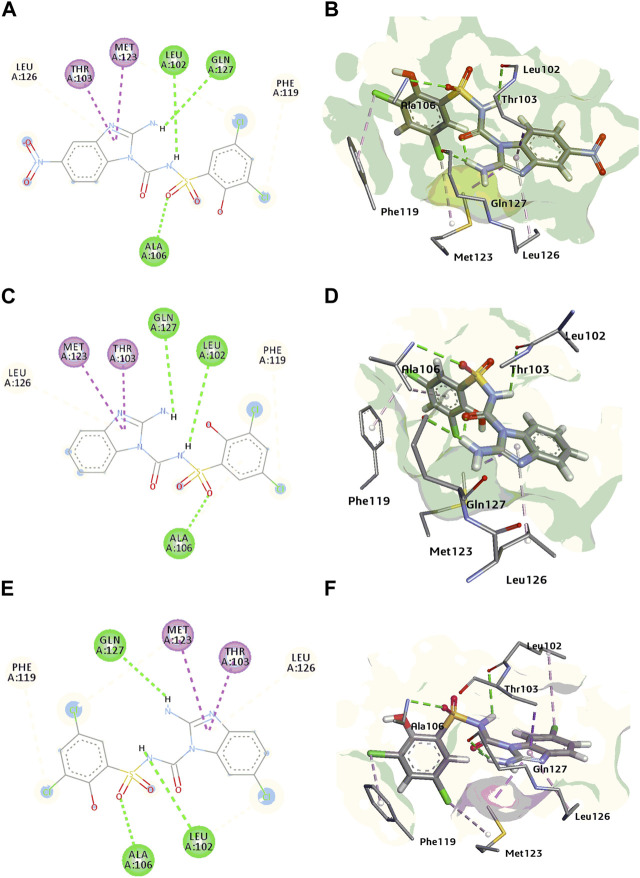
**(A, B)** 2D and 3D binding interactions of P2Y1 receptor inhibitor 1a **(C, D)** 2D and 3D binding interactions of P2Y1 receptor inhibitor 1e **(E, F)** 2D and 3D binding interactions of P2Y1 receptor inhibitor 1h.

Molecular docking of potent compounds **1a, 1e** and **1h** was done in reported allosteric binding pocket of BPTU. The possible binding interactions for compound **1a** with P2Y1 illustrated in Figure 8A, B. Leu02 and Ala106 show hydrogen bonding interaction with sulfonylurea linkage and oxygen atom of (SO2) group of compound 1a, similarly Leu126, Phe119, Thr103 and Met123 show hydrophobic interactions.

In case of compound **1e,** Leu102, Gln127 and Ala106 showed H-bond with NH of sulfonylurea, amino group of benzimidazole nucleus and oxygen atom of the sulphonamide group of compound **1e**. Met123, Thr103, Phe119 and Leu126 amino acid residues surrounded the compound with hydrophobic interactions Figures 8C, D.

Compound **1h**, the most potent compound of the series binds with the allosteric pocket of p2y1 receptor with the binding energy of −6.02 kcal/mol. Figures 8E, F explained the interaction of with P2Y1 receptor. Gln127 showed hydrogen bond with NH of the benzimidazole nucleus oxygen atom of the sulphonamide showed H-bond interaction with the residue Ala106 while the Leu102 showed the hydrogen bonding with NH of the sulphonyl urea linkage of **1h**. Leu102 also showed the hydrophobic interaction with the chloro group of the benzimidazole nucleus. Moreover, benzimidazole nucleus was surrounded by hydrophobic pocket of Met123, Thr103 and Leu126 residues. Phe119 was involved in the alkyl interaction with compound **1h**.

The leading interaction that were observed in standard antagonist (BPTU) were also observed in our synthesized compounds. There are a few interactions found to be conserved on P2Y1R-ligands **1a, 1e** and **1h** complexes at the amino acid residues such as Leu102, Ala106, Phe119, Thr103, Met123, and Leu126. These observations suggest that these ligands have the potential to bind with the P2Y1 receptor. Binding energy of synthesized potent compounds were less than the potent BPTU antagonist because of the replacement of urea linkage with amine group contained within five membered heterocyclic rings (1a-y), thus suggesting that only one H-bond with the backbone of L102 is needed to bind at the P2Y1R.

## 4 Discussion

Over-expression of P2Y1 receptors have been reported to be involved in pathophysiology of various disorders including inflammation, thrombosis, pain, osteoporosis, bladder abnormalities as well as neurological dysfunctions. There are limited antagonists of P2Y1 receptors available. Therefore, there is a need to synthesize new class of potent P2Y1 receptor antagonists with improved pharmacokinetic properties. Based on the presence of urea group in BPTU and suramin, sulphonylurea in Elinogrel, carboxamide group in SAR 216471 hydrochloride and benzimidazole nucleus in KCB77033 of previously reported compounds, shown in Figure 1, a new class of compounds (**1a-y**) was synthesized as P2Y1 receptor antagonists in this study.

Cell viability assay was performed to evaluate the cytotoxic nature of the effective compounds and lower the possible effects of calcium influx that might be produced by cell toxicity producing false positive results. None of the sulfonylurea derivatives were identified as cell toxic, when the synthesised molecules were investigated with dose up to 50 μM. As a result, it was believed that any inhibitory impact seen by calcium influx assays was unlikely to be caused by the cytotoxicity of synthesized series (**1a-y**).

Expression and functional analysis of P2Y1, P2Y2, P2Y4 and P2Y6 receptors in h-1321N1 astrocytoma cell line was performed through Real time-PCR and calcium mobilization assay. Products of RT-PCR were run on 2% agarose gel which resulted in the appearance of strong bands between 100 and 200 bp Furthermore, functional assay i.e., calcium mobilization assay demonstrated the expression of receptor proteins lines by a sudden increase in fluorescence signals as compared to a cell line that had not been transfected which produced no fluorescence signal.

The effectiveness of the synthesised compounds were assessed through fluorescently based calcium mobilization assay which follow the standard methodology i.e., the Ca^+2^ sensitive acetoxymethyl (AM) ester of Fura-2 fluorescence dye was injected into stably transfected P2YRs- 1321N1 astrocytoma cells. Four P2YR subtypes i.e., turkey P2Y1R, human P2Y2R and P2Y4R, and rat P2Y6 receptors were used in this study. Gq-protein coupled receptors, such as P2Y receptor upon ligand activation., ATP, ADP, UDP and UTP causes abrupt rise in the intracellular Ca^+2^ concentration as a result of the release of the fluorescence light by the Fura-2 AM dye. Synthetic substances that act as P2Y receptor antagonists prevent the increase in the intensity of the fluorescence which is measured via a fluorescent microplate reader. Among all the synthesized derivatives, compound **1a, 1e** and **1h** were found potent derivatives exhibited strong inhibition of P2Y1 receptor. Compound **1h** exhibited more potential as compared to **1a** and **1e** with an IC_50_ values of 0.19 ± 0.04, 0.37 ± 0.02 and 0.34 ± 0.01 µM. The presence of electronegative Cl group resulted in the improved inhibition of the targeted P2Y1 receptor. The detailed structure activity relationship was described in detail in result section.

The improved aqueous solubility of compound **1h** provides a way to evaluate their functional profile *in vivo*. In silico studies of the potent compounds were also performed which support *in vitro* results. Compound **1h** showed considerable interaction comparable with BPTU at the allosteric site which is located on the outer hydrophobic surface of the transmembrane (TM) bundles of the receptor.

## 5 Conclusion

All the synthesized compounds based on benzimidazole and sulfonylurea were synthesized and characterized through spectroscopic techniques and evaluated for their inhibitory potential against P2Y receptor subtypes. The synthesized compounds exhibited less cytotoxicity compared to the reference compound, and most of them displayed good to excellent inhibitory activity. Compound **1h** with 3,5-diCl, 2-OH substitution showed the highest potency with an IC_50_ value of 0.19 μM against the P2Y1 receptor, which is comparable to the non-selective inhibitor suramin. Additionally, compounds **1a, 1e**, and **1f** showed excellent inhibition towards P2Y1 receptor. Compound **1h** also depicted inhibitory potential against P2Y2 and P2Y4 receptors but was inactive against P2Y6. The aqueous solubility of compound **1h** was significantly higher than the reported BPTU antagonist, indicating its potential for *in vivo* functional activity. Furthermore, computational studies revealed that compounds **1a, 1e,** and **1h** formed complexes with P2Y1R-ligands that interacted with amino acid residues Leu102, Ala106, Phe119, and Met123. These results suggest that the P2Y1 receptor may bind with these ligands. Overall, the synthesized benzimidazole-based sulfonylurea derivatives represent a promising group of compounds for further development as P2Y receptor subtype inhibitors.

## Data Availability

The raw data supporting the conclusion of this article will be made available by the authors, without undue reservation.

## References

[B1] Abbracchio M. P. Burnstock G. Boeynaems J. M. Barnard E. A. Boyer J. L. Kennedy C. (2006). International union of Pharmacology LVIII: Update on the P2Y G protein-coupled nucleotide receptors: From molecular mechanisms and pathophysiology to therapy. Pharmacol. Rev. 58 (3), 281–341. 10.1124/pr.58.3.3 16968944 PMC3471216

[B2] Alves M. Beamer E. Engel T. (2018). The metabotropic purinergic P2Y receptor family as novel drug target in epilepsy. Front. Pharmacol. 9, 193. 10.3389/fphar.2018.00193 29563872 PMC5851315

[B3] Anwar S. Pons V. Rivest S. (2020). Microglia purinoceptor P2Y6: An emerging therapeutic target in CNS diseases. Cells 9, 1595. 10.3390/cells9071595 32630251 PMC7407337

[B4] Bach P. Bostr J. Brickmann K. Van Giezen J. J. J. Groneberg R. D. Harvey D. M. (2013). Synthesis, structure–property relationships and pharmacokinetic evaluation of ethyl 6-aminonicotinate sulfonylureas as antagonists of the P2Y12 receptor. Eur. J. Med. Chem. 65, 360–375. 10.1016/j.ejmech.2013.04.007 23747805

[B5] Bae J. Kim Y. O. Han X. Yoon M. H. Kim W. M. Kim Y. C. (2022). Synthesis and structure–activity relationship studies of benzimidazole-4, 7-dione-Based P2X3 receptor antagonists as novel anti-nociceptive agents. Molecules 27, 1337. 10.3390/molecules27041337 35209126 PMC8877008

[B6] Bano S. Shabir G. Saeed A. Ul-Hamid A. Alharthy R. D. Iqbal J. (2021). Synthesis, characterization and biological evaluation of indomethacin derived thioureas as purinergic (P2Y1, P2Y2, P2Y4, and P2Y6) receptor antagonists. Bioorg. Chem. 116, 105378. 34601296 10.1016/j.bioorg.2021.105378

[B7] Discovery Studio Visualizer DS (2017). Dassault Systemes BIOVIA Discovery Studio Modeling Environment. San Diego, CA: Dassault Systemes.

[B8] Boldron C. Besse A. Bordes M. F. Tissandié S. Yvon X. Gau B. (2014). N-[6-(4-Butanoyl-5-methyl-1 H-pyrazol-1-yl) pyridazin-3-yl]-5-chloro-1-[2-(4-methylpiperazin-1-yl)-2-oxoethyl]-1 H-indole-3-carboxamide (SAR216471), a novel intravenous and oral, reversible, and directly acting P2Y12 antagonist. J. Med. Chem. 57, 7293–7316. 10.1021/jm500588w 25075638

[B9] Burnstock G. (2006). Pathophysiology and therapeutic potential of purinergic signaling. Pharmacol. Rev. 58, 58–86. 10.1124/pr.58.1.5 16507883

[B10] Burnstock G. (2017). Purinergic signalling: Therapeutic developments. Front. Pharmacol. 8, 661. 10.3389/fphar.2017.00661 28993732 PMC5622197

[B11] Chen S. Shenk T. Nogalski M. T. (2019). P2Y2 purinergic receptor modulates virus yield, calcium homeostasis, and cell motility in human cytomegalovirus-infected cells. Proc. Natl. Acad. Sci. 116, 18971–18982. 10.1073/pnas.1907562116 31481624 PMC6754545

[B12] Erb L. Weisman G. A. (2012). Coupling of P2Y receptors to G proteins and other signaling pathways. Wiley Interdiscip. Rev. Membr. Transp. Signal. 1, 789–803. 10.1002/wmts.62 25774333 PMC4358762

[B13] Filtz T. M. Li Q. Boyer J. L. Nicholas R. A. Harden T. K. (1994). Expression of a cloned P2Y purinergic receptor that couples to phospholipase C. Mol. Pharmacol. 46, 8–14. PMID: 8058061. 8058061

[B14] Gacasan S. B. Baker D. L. Parrill A. L. (2017). G protein-coupled receptors: The evolution of structural insight. AIMS Biophys. 4, 491. 10.3934/biophy.2017.3.491 29951585 PMC6018013

[B15] Govindan S. Taylor C. W. (2012). P2Y receptor subtypes evoke different Ca ^2+^ signals in cultured aortic smooth muscle cells. Purinergic Signal 8, 763–777. 10.1007/s11302-012-9323-6 22767215 PMC3486169

[B16] Hechler B. Gachet C. (2011). P2 receptors and platelet function. Purinergic Signal 7, 293. 10.1007/s11302-011-9247-6 21792575 PMC3166986

[B17] Hechler B. Gachet C. (2015). Purinergic receptors in thrombosis and inflammation. Arterioscler. Thromb. Vasc. Biol. 35, 2307–2315. 10.1161/atvbaha.115.303395 26359511

[B18] Hillmann P. Ko G. Y. Spinrath A. Raulf A. von Kügelgen I. Wolff S. C. (2009). Key determinants of nucleotide-activated G protein-coupled P2Y2 receptor function revealed by chemical and pharmacological experiments, mutagenesis and homology modeling. J. Med. Chem. 52, 2762–2775. 10.1021/jm801442p 19419204

[B19] Hoffmann C. Moro S. Nicholas R. A. Harden T. K. Jacobson K. A. (1999). The role of amino acids in extracellular loops of the human P2Y1 receptor in surface expression and activation processes. J. Biol. Chem. 274, 14639–14647. 10.1074/jbc.274.21.14639 10329657 PMC3449168

[B20] Jacobson K. A. Ivanov A. A. de Castro S. Harden T. K. Ko H. (2009). Development of selective agonists and antagonists of P2Y receptors. Purinergic Signal 5, 75–89. 10.1007/s11302-008-9106-2 18600475 PMC2721770

[B21] Kassack M. U. Höfgen B. Lehmann J. Eckstein N. Quillan J. M. Sadee W. (2002). Functional screening of G protein–coupled receptors by measuring intracellular calcium with a fluorescence microplate reader. J. Biomol. Screen. 7, 233–246. 10.1177/108705710200700307 12097186

[B22] Le H. T. T. Rimpilainen T. Konda Mani S. Murugesan A. Yli-Harja O. Candeias N. R. (2019). Synthesis and preclinical validation of novel P2Y1 receptor ligands as a potent anti-prostate cancer agent. Sci. Rep. 9, 1–11. 10.1038/s41598-019-55194-8 31831761 PMC6908675

[B23] Li W. H. Qiu Y. Zhang H. Q. Liu Y. You J. F. Tian X. X. (2013). P2Y2 receptor promotes cell invasion and metastasis in prostate cancer cells. Br. J. Cancer. 109, 1666–1675. 10.1038/bjc.2013.484 23969730 PMC3776994

[B24] Mahmood A. Munir R. Zia-ur-Rehman M. Javid N. Shah S. J. A. Noreen L. (2021). Synthesis of sulfonamide tethered (hetero) aryl ethylidenes as potential inhibitors of P2X receptors: A promising way for the treatment of pain and inflammation. ACS Omega 6, 25062–25075. 10.1021/acsomega.1c04302 34604685 PMC8482771

[B25] Oestreich J. H. (2010). Elinogrel, a reversible P2Y12 receptor antagonist for the treatment of acute coronary syndrome and prevention of secondary thrombotic events. Curr. Opin. Investig. Drugs 11, 340–348. 20178048

[B26] Parravicini C. Ranghino G. Abbracchio M. P. Fantucci P. (2008). GPR17: Molecular modeling and dynamics studies of the 3-D structure and purinergic ligand binding features in comparison with P2Y receptors. BMC Bioinform 9, 1–19. 10.1186/1471-2105-9-263 PMC2443813 18533035

[B27] Peng J. Zhao L. Wang L. Chen H. Qiu Y. Wang J. (2018). Design, synthesis, and biological evaluation of 2-(phenoxyaryl)-3-urea derivatives as novel P2Y1 receptor antagonists. Eur. J. Med. Chem. 158, 302–310. 10.1016/j.ejmech.2018.09.014 30223118

[B28] Pi Z. Sutton J. Lloyd J. Hua J. Price L. Wu Q. (2013). Bioorg. Med. Chem. Lett. 23, 4206–4209. 10.1016/j.bmcl.2013.05.025 23743287

[B29] Rafehi M. Burbiel J. C. Attah I. Y. Abdelrahman A. Müller C. E. (2017). Synthesis, characterization, and *in vitro* evaluation of the selective P2Y 2 receptor antagonist AR-C118925. Purinergic Signal 13, 89–103. 10.1007/s11302-016-9542-3 27766552 PMC5334202

[B30] Ruel R. L’Heureux A. Thibeault C. Daris J. P. Martel A. Price L. A. (2013). New azole antagonists with high affinity for the P2Y1 receptor. Bioorg. Med. Chem. Lett. 23, 3519–3522. 10.1016/j.bmcl.2013.04.041 23668989

[B31] Sauer R. El-Tayeb A. Kaulich M. Müller C. E. (2009). Synthesis of uracil nucleotide analogs with a modified, acyclic ribose moiety as P2Y2 receptor antagonists. Bioorg. Med. Chem. 17, 5071–5079. 10.1016/j.bmc.2009.05.062 19523835

[B32] Uddin M. S. Hauser M. Naider F. Becker J. M. (2016). The N-terminus of the yeast G protein-coupled receptor Ste2p plays critical roles in surface expression, signaling, and negative regulation. Biochim. Biophys. Acta Biomembr. 1858, 715–724. 10.1016/j.bbamem.2015.12.017 PMC4779653 26707753

[B33] Ullah S. El-Gamal M. I. El-Gamal R. Pelletier J. Sevigny J. Shehata M. K. (2021). Synthesis, biological evaluation, and docking studies of novel pyrrolo [2, 3-b] pyridine derivatives as both ectonucleotide pyrophosphatase/phosphodiesterase inhibitors and antiproliferative agents. Eur. J. Med. Chem. 217, 113339. 10.1016/j.bioorg.2020.103783 33744686

[B34] Ullmann H. Meis S. Hongwiset D. Marzian C. Wiese M. Nickel P. B. (2005). Synthesis and structure− activity relationships of suramin-derived P2Y11 receptor antagonists with nanomolar potency. J. Med. Chem. 48, 7040–7048. 10.1021/jm050301p 16250663

[B35] Xu P. Feng X. Luan H. Wang J. Ge R. Li Z. (2018). Current knowledge on the nucleotide agonists for the P2Y2 receptor. Bioorg. Med. Chem. 26, 366–375. 10.1016/j.bmc.2017.11.043 29254895

[B36] Zhang D. Gao Z. G. Zhang K. Kiselev E. Crane S. Wang J. W. (2015). Two disparate ligand-binding sites in the human P2Y1 receptor. Nature 520, 317–321. 10.1038/nature14287 25822790 PMC4408927

