# Supplementary material for: Synthesis, structure-activity relationships and biological evaluation of benzimidazole derived sulfonylurea analogues as a new class of antagonists of P2Y1 receptor
Source: Front Pharmacol. 2023 May 26;14:1217315. doi: 10.3389/fphar.2023.1217315 (PMC10250618; doi:10.3389/fphar.2023.1217315)
Supplement: Supplementary file 1 [file Table1.docx]

Supplementary Material

Synthesis, Structure-Activity Relationships and Biological Evaluation of Benzimidazole derived Sulfonylurea analogues as a new class of Antagonists of P2Y1 Receptor

**Sehrish Bano ^1,2^, Zahid Hussain^1^, Peter Langer^3^, Gary A. Weisman^4^, Jamshed Iqbal ^1,2*^**

^1^Centre for Advanced Drug Research, COMSATS University Islamabad, Abbottabad Campus, Abbottabad, 22060, Pakistan

^2^Department of Pharmacy, COMSATS University Islamabad, Abbottabad Campus, Abbottabad, 22060, Pakistan

^3^Institut für Chemie, Universität Rostock, Albert Einstein Str. 3a, 18059 Rostock, Germany

^4^Department of Biochemistry, University of Missouri-Columbia, 540E Life Sciences Center, Columbia, MO 65211, USA

*** Correspondence:**Prof. Dr. Jamshed Iqbal

drjamshed@cuiatd.edu.pk /jamshediqb@googlemail.com

1. **Representative ^1^ H and ^13^ C NMR Spectra:**

NMR (including ^1^ H and ^13^ C NMR) spectra for representative compounds are given below

#

**^1^H NMR**

**Supplementary Figure 1.** 1H NMR spectrum for **1a**

#
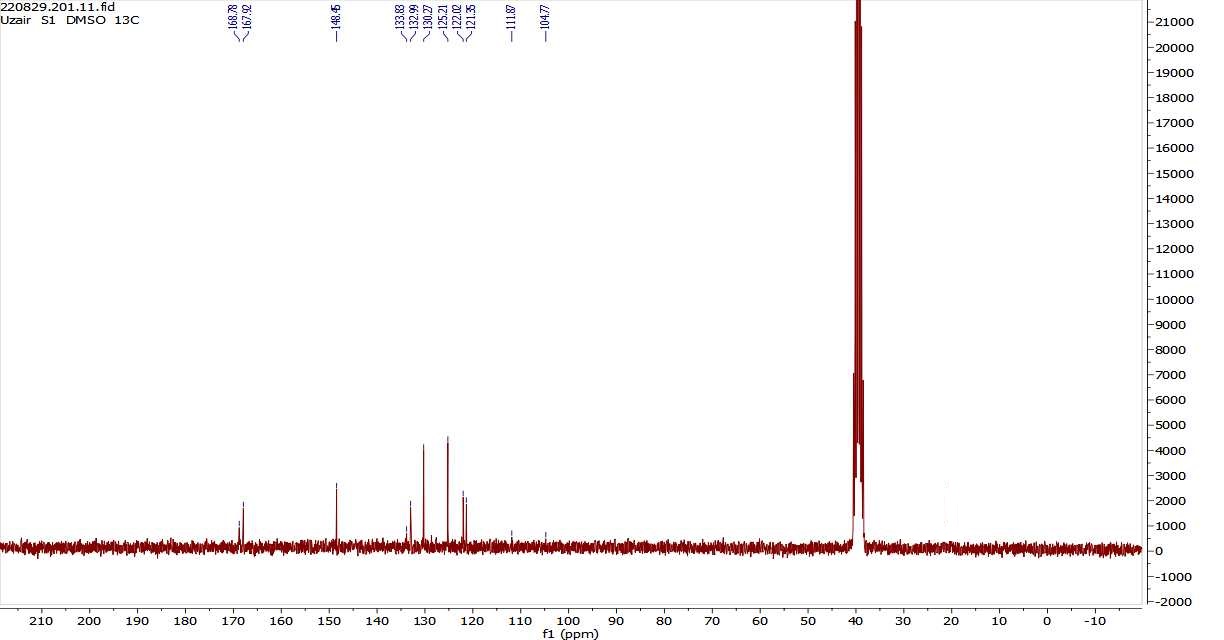


**^13^C NMR**

# Supplementary Figure 2. ^13^C NMR spectrum for 1a

#

**^1^H NMR**

**Supplementary Figure 3.** 1H NMR spectrum for **1c**

#
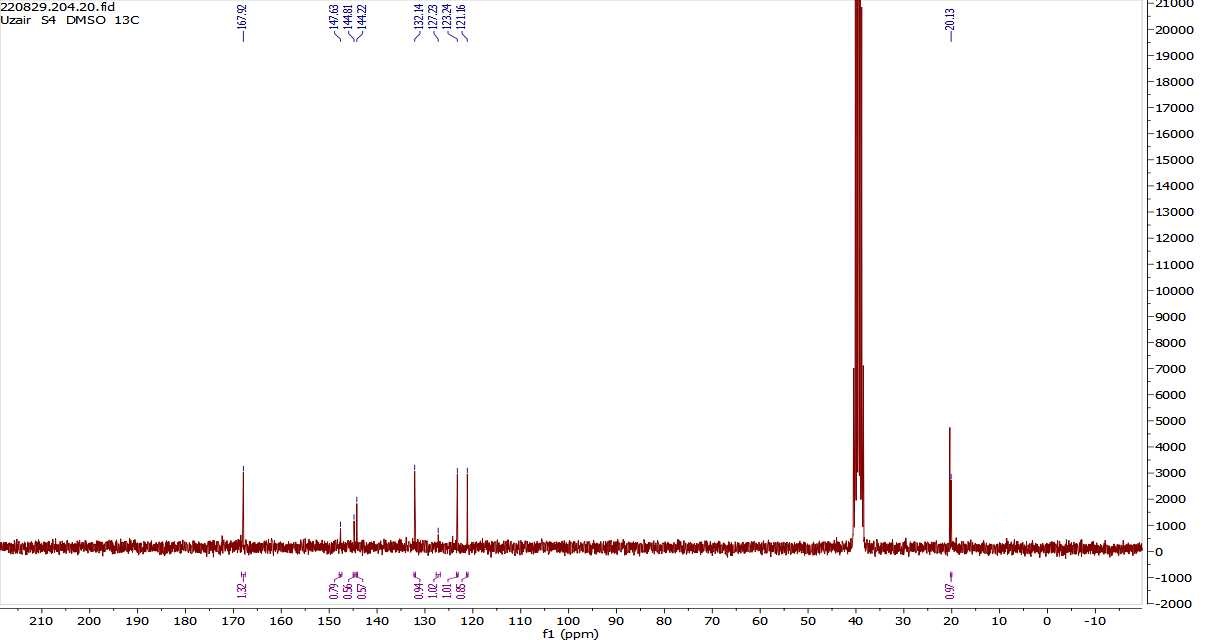


**^13^C NMR**

# Supplementary Figure 4. ^13^C NMR spectrum for 1c

#
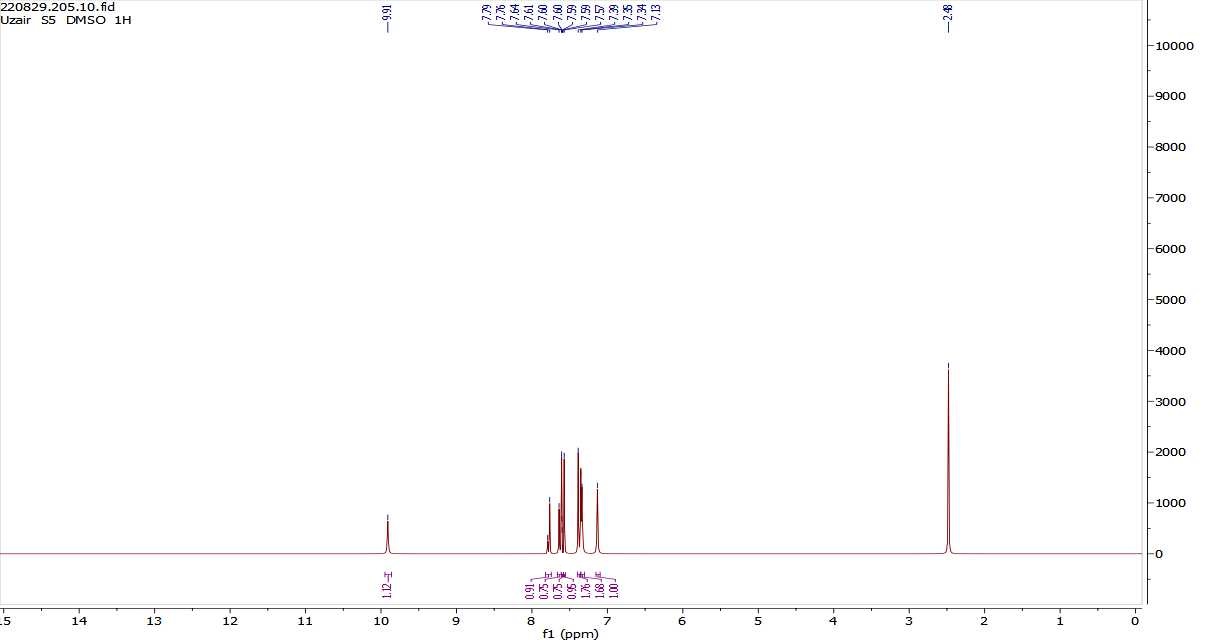


**^1^H NMR**

# Supplementary Figure 5. ^1^H NMR spectrum for 1d

# Supplementary Figure 6. ^1^H NMR spectrum for 1e

**^1^H NMR**

#

**^1^H NMR**

**Supplementary Figure 6.** 1H NMR spectrum for **1f**

#
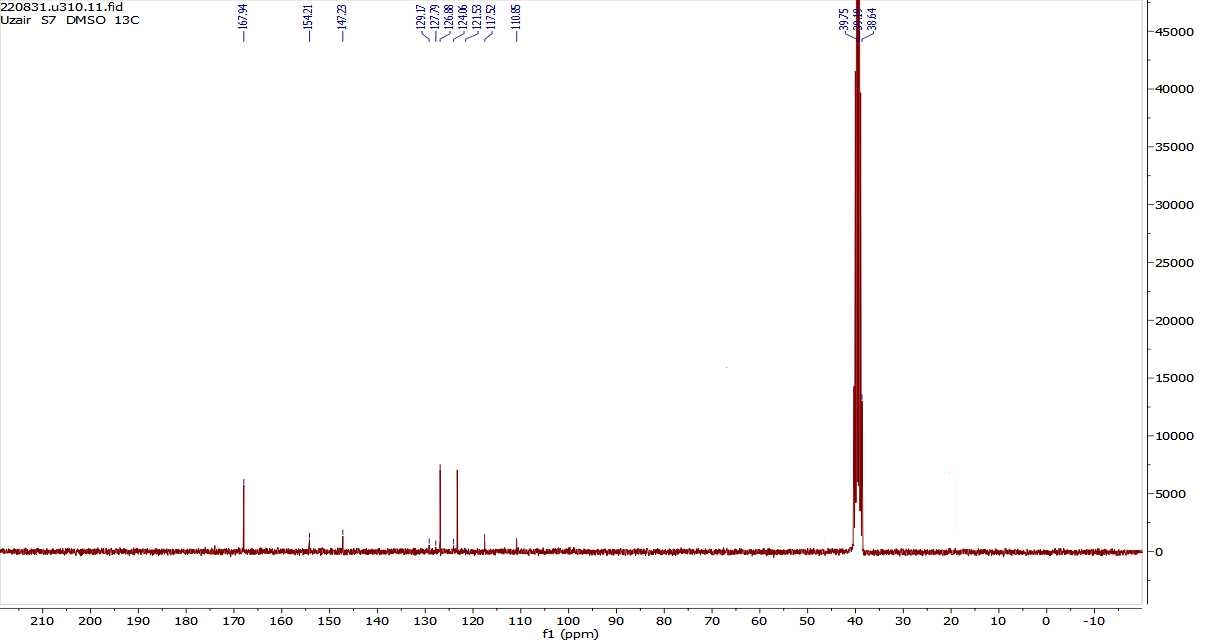


**^13^C NMR**

# Supplementary Figure 8. ^13^C NMR spectrum for 1f

# Supplementary Figure 9. ^1^H NMR spectrum for 1g

**^1^H NMR**

#

**^1^H NMR**

**Supplementary Figure 10.** 1H NMR spectrum for **1h**

#
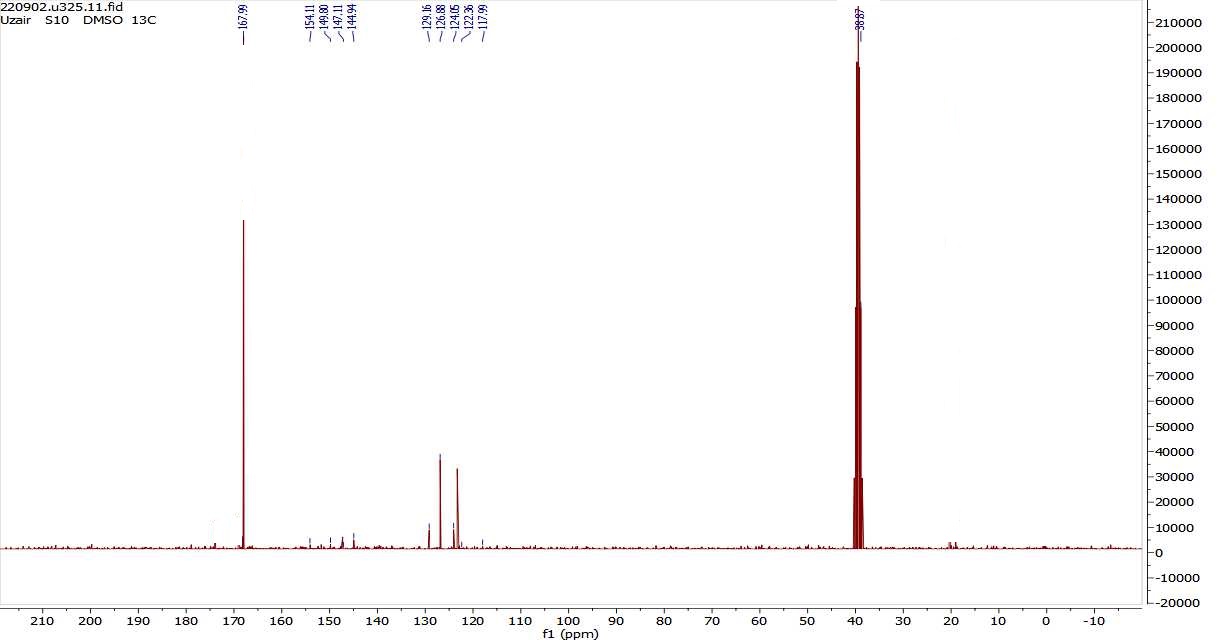


**^13^C NMR**

# Supplementary Figure 11. ^13^C NMR spectrum for 1h

#

# Supplementary Figure 12. ^1^H NMR spectrum for 1i

#
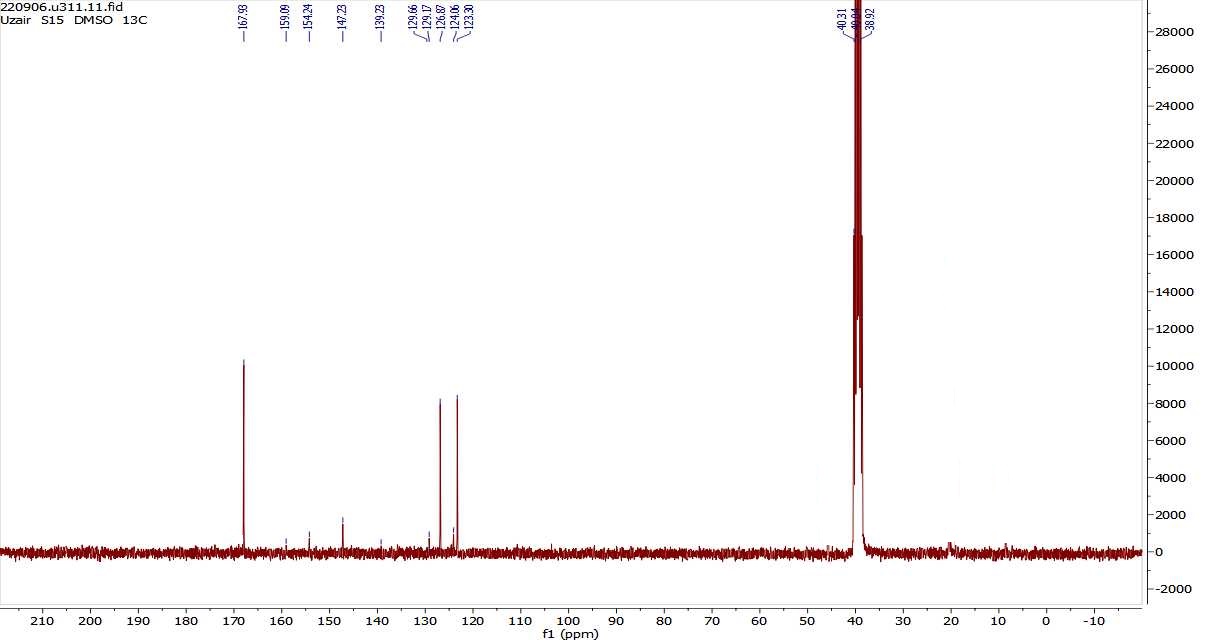


**^1^H NMR**

# Supplementary Figure 13. ^13^C NMR spectrum for 1k

#

**^13^C NMR**

# Supplementary Figure 14. ^1^H NMR spectrum for 1k

#

**^1^H NMR**

# Supplementary Figure 15. ^1^H NMR spectrum for 1j

# Supplementary Figure 16. ^1^H NMR spectrum for 1l

**^1^H NMR**

#
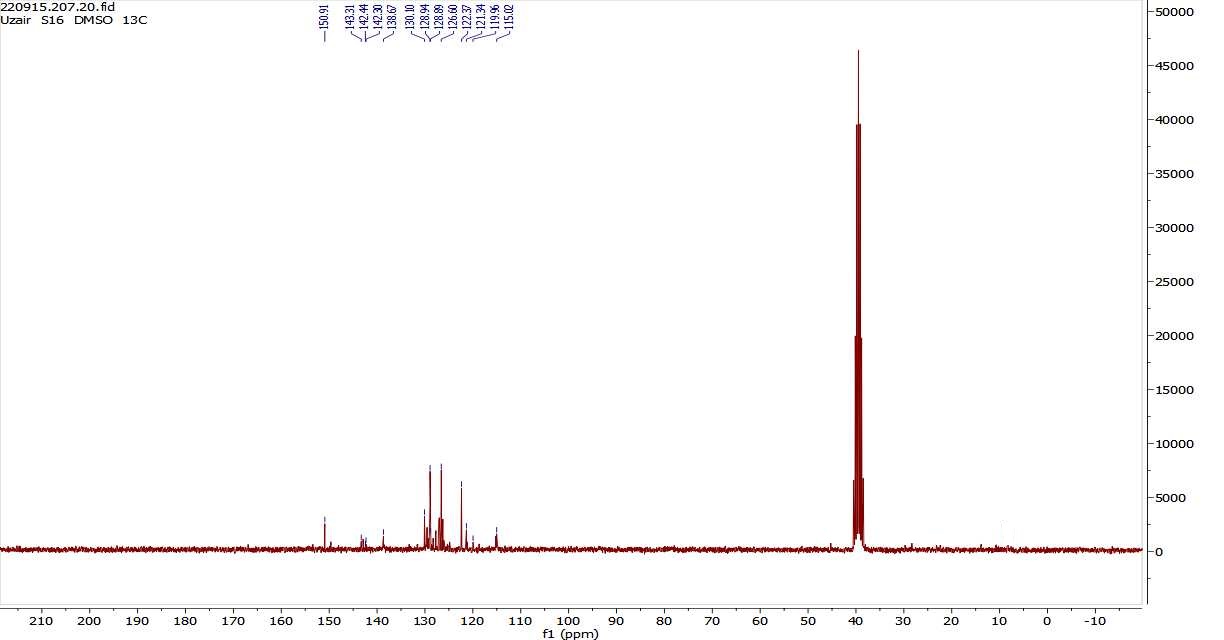


**^13^C NMR**

**Supplementary Figure 17** ^13^C NMR spectrum for **1l**

# Supplementary Figure 18. ^1^H NMR spectrum for 1m

**^1^H NMR**

#
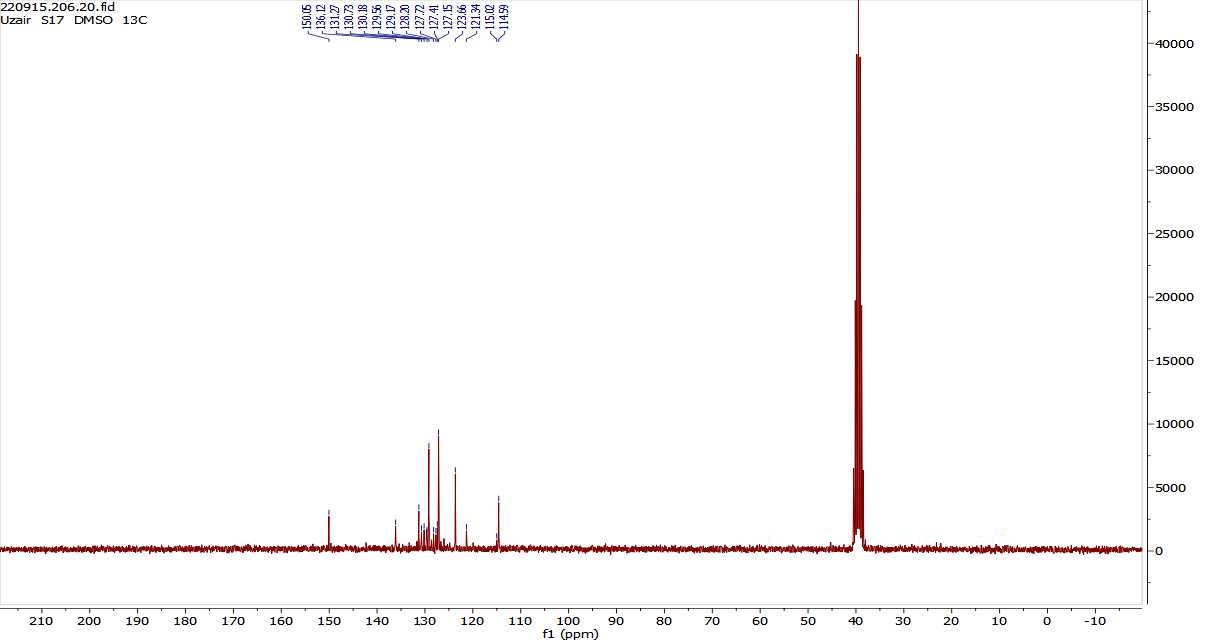


**^13^C NMR**

**Supplementary Figure 19.** ^13^C NMR spectrum for **1m**

#

**^1^H NMR**

**Supplementary Figure 20.** 1H NMR spectrum for **1n**

#
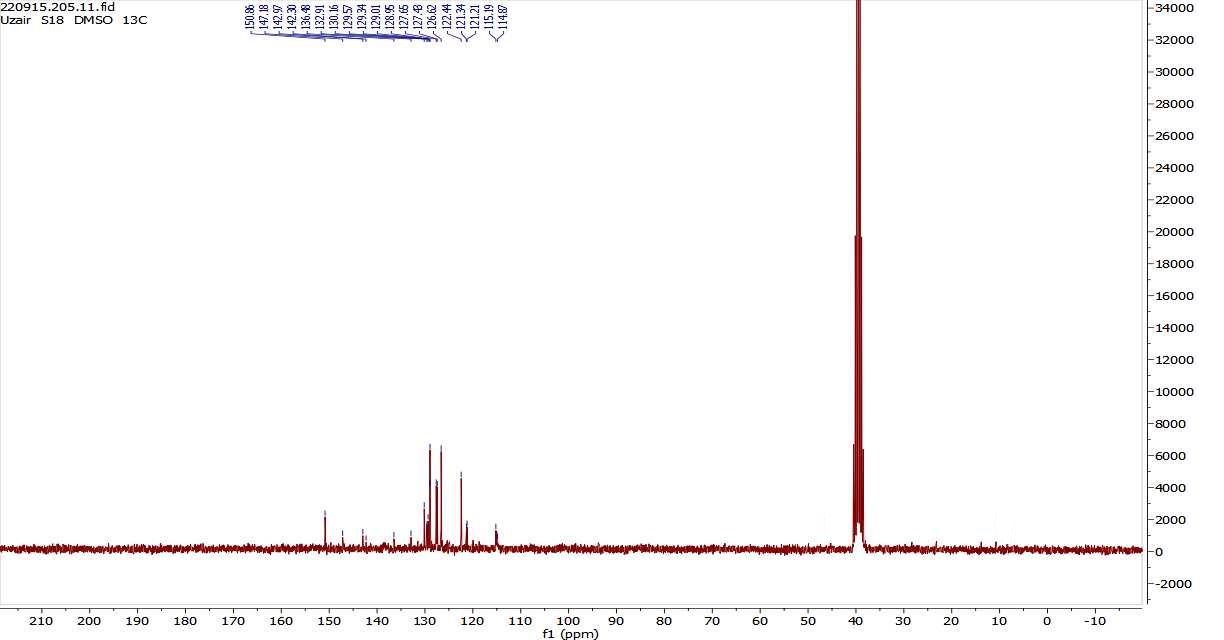


**^13^C NMR**

**Supplementary Figure 21.** ^13^C NMR spectrum for **1n**

#

**^1^H NMR**

**Supplementary Figure 22.** ^1^H NMR spectrum for **1o**

#

**^13^C NMR**

**Supplementary Figure 23.** ^13^C NMR spectrum for **1o**

#

**^1^H NMR**

**Supplementary Figure 24.** ^1^H NMR spectrum for **1p**

#
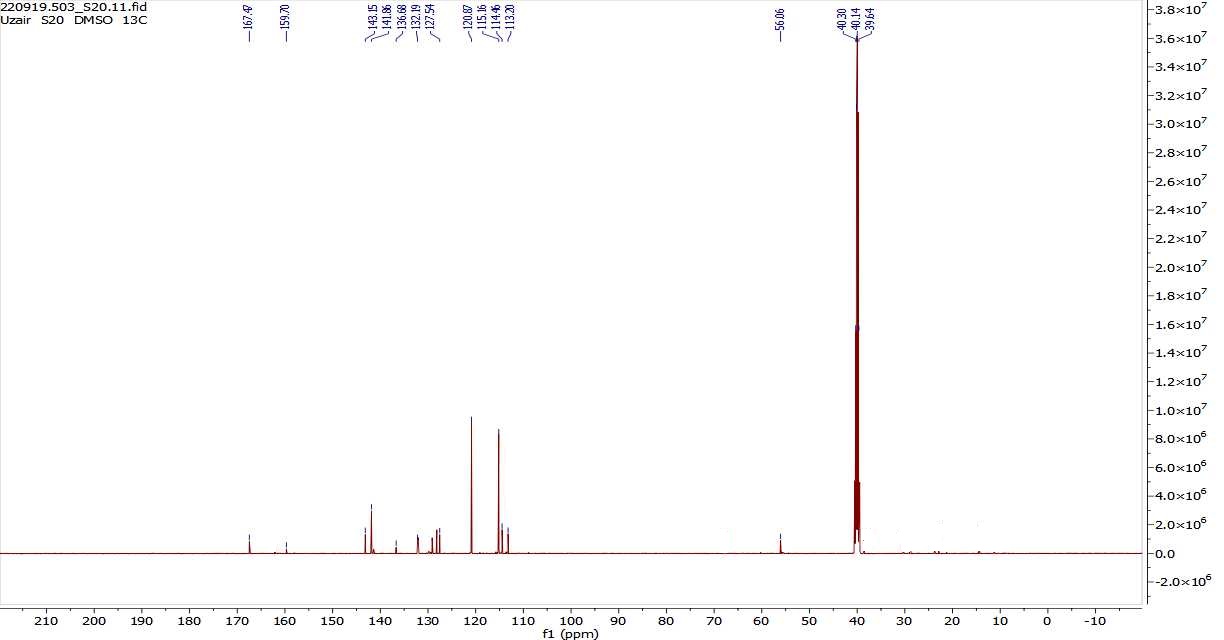


**^13^C NMR**

**Supplementary Figure 25.** ^13^C NMR spectrum for **1p**

#

**^1^H NMR**

**Supplementary Figure 26.** ^1^H NMR spectrum for **1q**

#

**^13^C NMR**

**Supplementary Figure 27.** ^13^C NMR spectrum for **1q**

#

**^1^H NMR**

**Supplementary Figure 28.** ^1^H NMR spectrum for **1r**

#
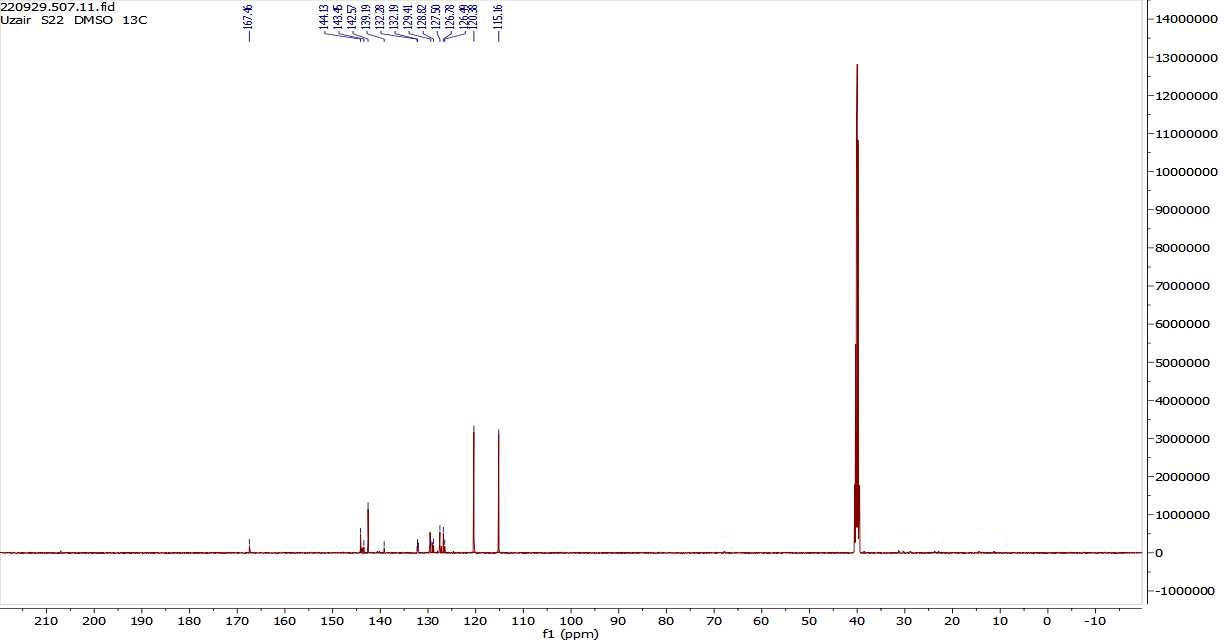


**^13^C NMR**

# Supplementary Figure 29. ^13^C NMR spectrum for 1r

#

**^1^H NMR**

**Supplementary Figure 30.** ^1^H NMR spectrum for **1s**

#
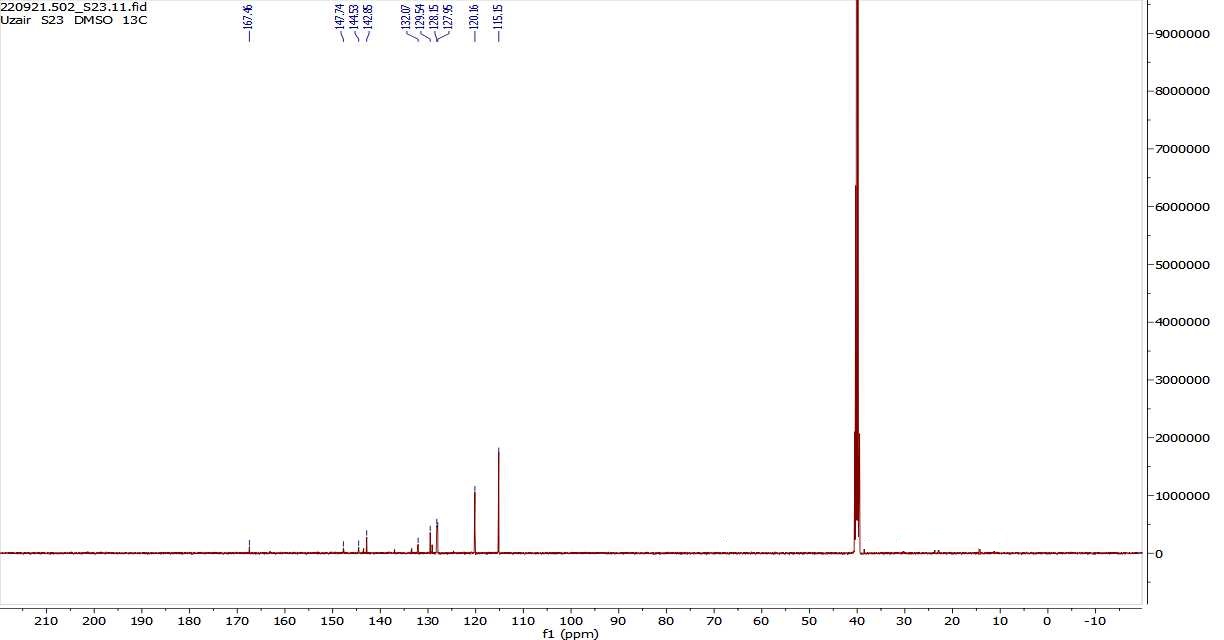


**^13^C NMR**

# Supplementary Figure 31. ^13^C NMR spectrum for 1s

#

**^1^H NMR**

**Supplementary Figure 32.** ^1^H NMR spectrum for **1t**

#
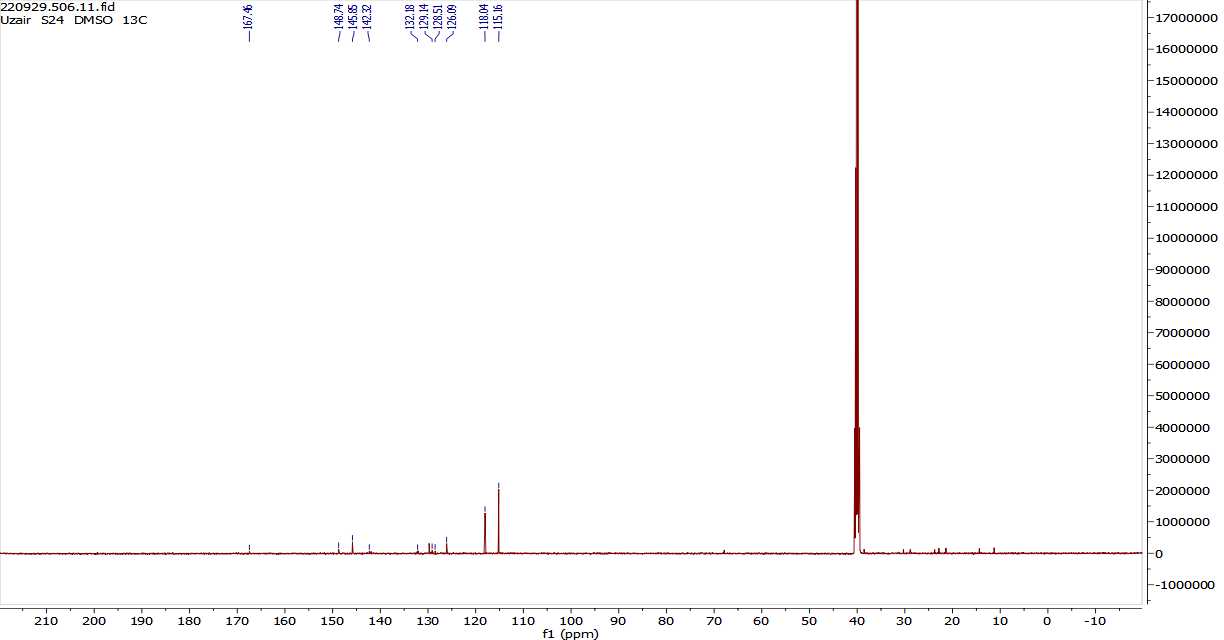


**^13^C NMR**

**Supplementary Figure 33.** ^1^H NMR spectrum for **1t**

#

**^1^H NMR**

# Supplementary Figure 34. ^1^H NMR spectrum for 1u

#
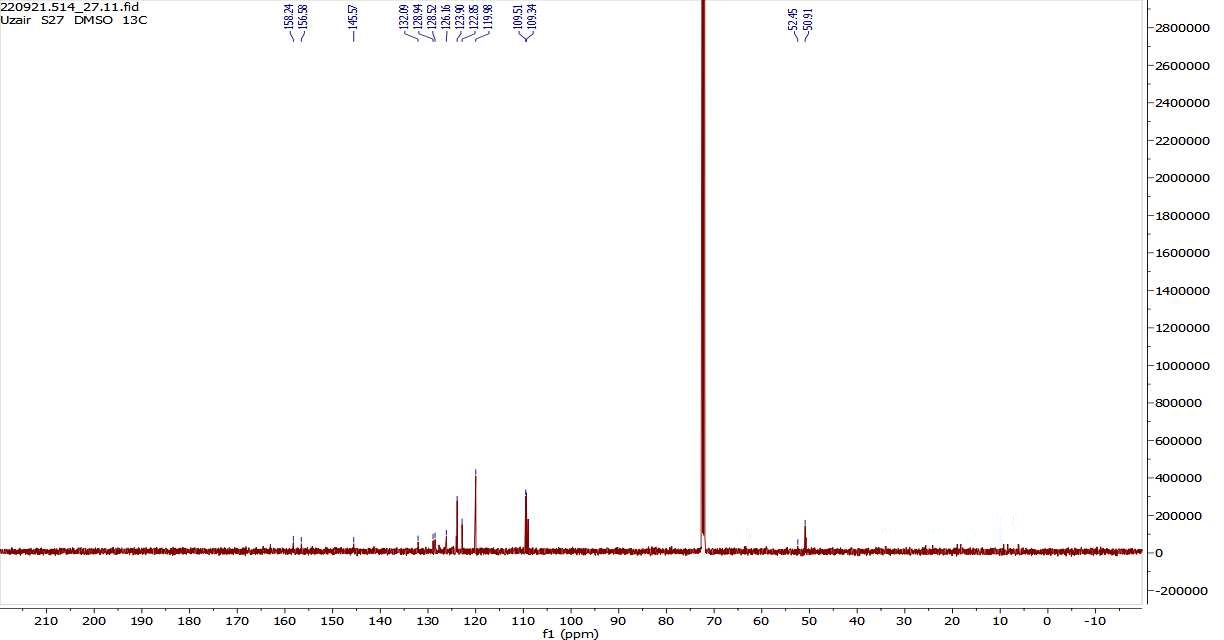


**^13^C NMR**

# Supplementary Figure 35. ^13^C NMR spectrum for 1u

#

**^1^H NMR**

**Supplementary Figure 36.** ^1^H NMR spectrum of 1**x**

#

**^13^C NMR**

# Supplementary Figure 37. ^13^C NMR spectrum for 1x

# Supplementary Table 1: IC_50_± SEM /% Inhibition for *h*-P2Y2 and *h*-P2Y4 receptors

| **Compound** | ***h*-P2Y2 IC_50_±SEM / % Inhibition** | **Graphs for IC_50_ Values (*h***-**P2Y2)** | **h-P2Y4 IC_50_±SEM/% Inhibition** | **Graphs for IC_50_ Values (*h*-P2Y4)** |
| --- | --- | --- | --- | --- |
| **1a** | 44% |  | 46% |  |
| **1b** | 38% |  | 18% |  |
| **1c** | 6.32 ± 1.10 | **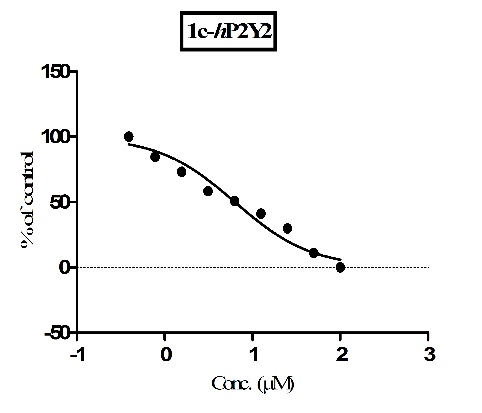** | 42% |  |
| **1d** | 41% |  | 48% |  |
| **1e** | 5.50 ± 1.21 | **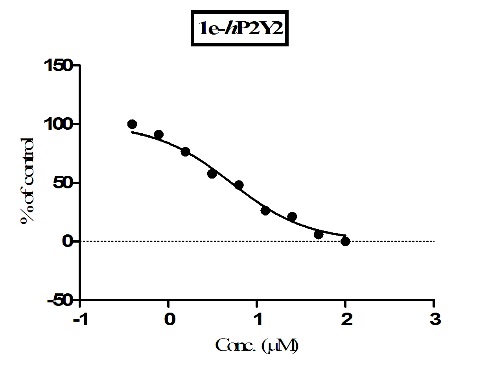** | 0.60 ± 0.01 | **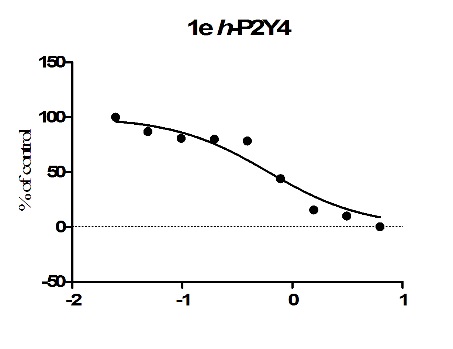** |
| **1f** | 8.41 ± 0.07 | **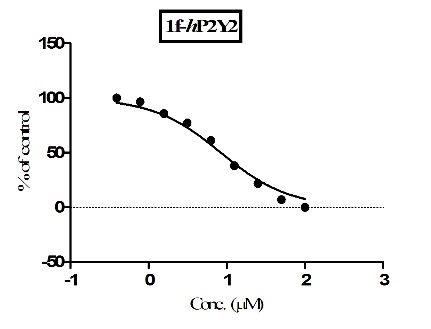** | 47% |  |
| **1g** | 33% |  | 13% |  |
| **1h** | 4.13 ± 1.02 | **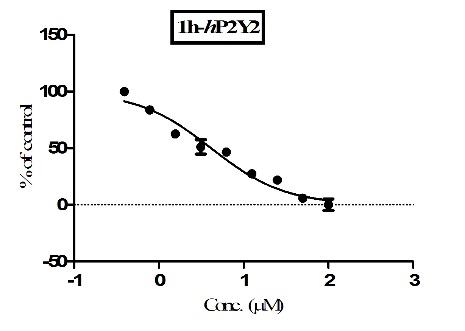** | 0.91 ± 0.04 | **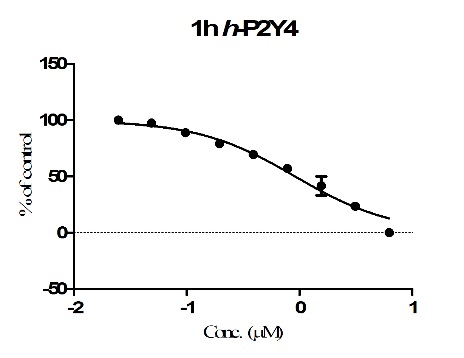** |
| **1i** | 15.66 ± 1.48 | **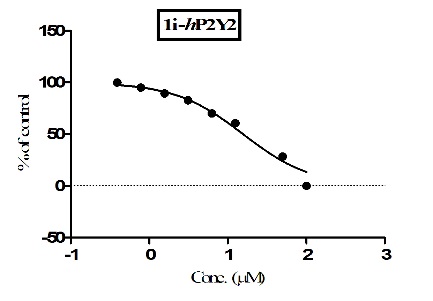** | 34% |  |
| **1j** | 32% |  | 42% |  |
| **1k** | 11.11 ± 0.94 | 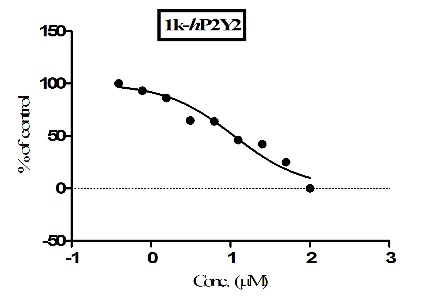 | 16.64 ± 1.12 | **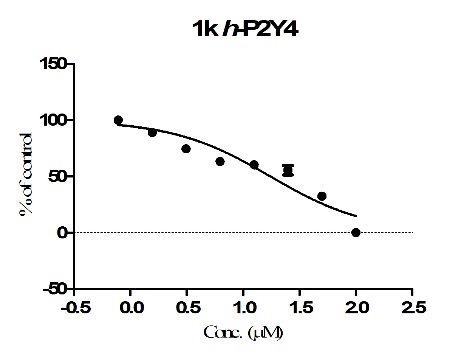** |
| **1l** | 34% |  | 22% |  |
| **1m** | 37% |  | 16% |  |
| **1n** | 21% |  | 19% |  |
| **1o** | 18.18 ± 0.77 | **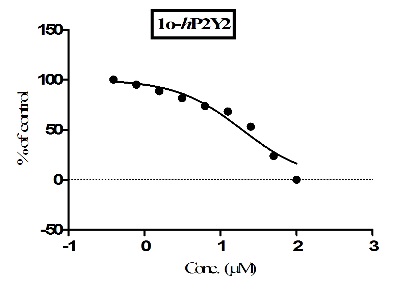** | 46% |  |
| **1p** | 12% |  | 20% |  |
| **1q** | 13.62 ± 1.97 | **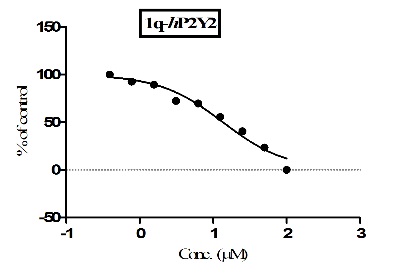** | 13% |  |
| **1r** | 47% |  | 22% |  |
| **1s** | 6.15 ± 1.08 | **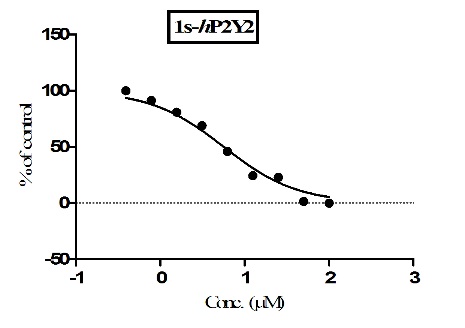** | 2.67 ± 0.07 | **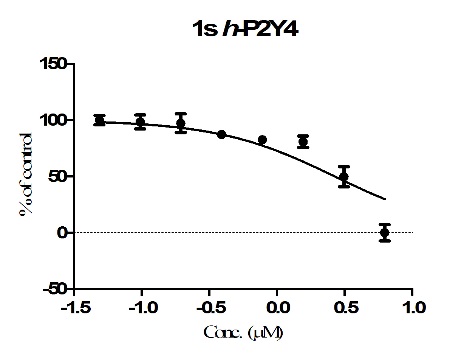** |
| **1t** | 42% |  | 26% |  |
| **1u** | 10% |  | 30% |  |
| **1v** | 19% |  | 23% |  |
| **1w** | 23% |  | 41% |  |
| **1x** | 38% |  | 29% |  |
| **1y** | 20% |  | 18% |  |

# Supplementary Table 2. IC_50_± SEM /% Inhibition for *r*-P2Y6

| **Compound** | ***r*-P2Y6 IC_50_±SEM / % Inhibition** | **Graphs for IC_50_ Values (*r*-P2Y6)** |
| --- | --- | --- |
| **1a** | 0.66 ± 0.04 | **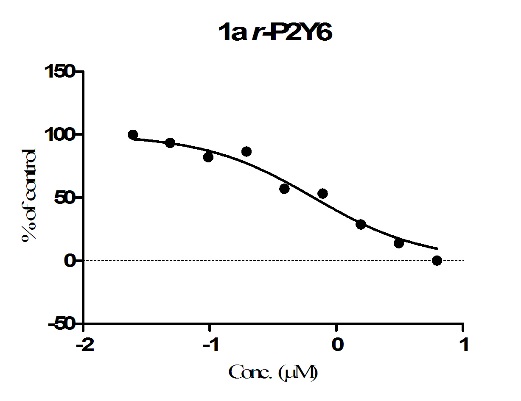** |
| **1b** | 21% |  |
| **1c** | 16.05 ± 0.59 | **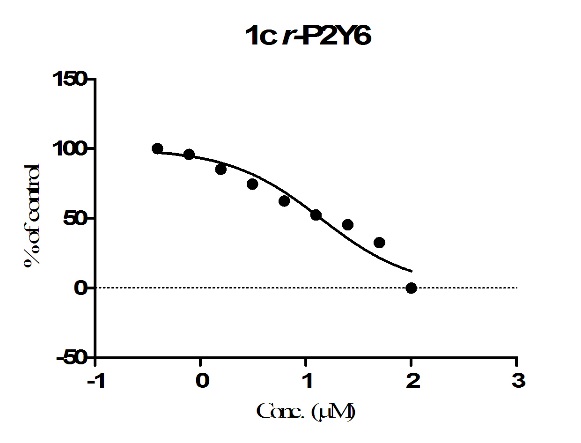** |
| **1d** | 39% |  |
| **1e** | 0.75 ± 0.08 | **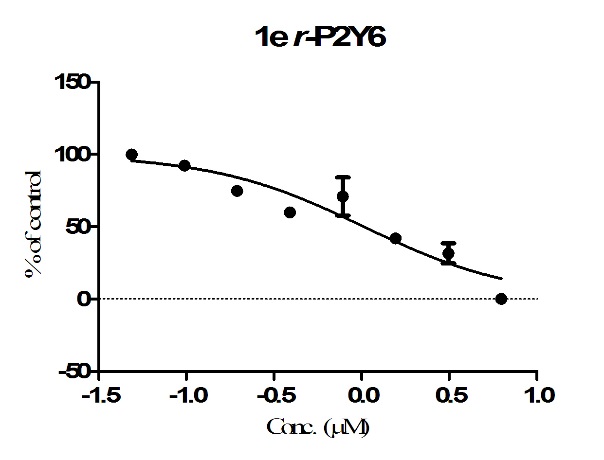** |
| **1f** | 9.30 ± 1.12 | **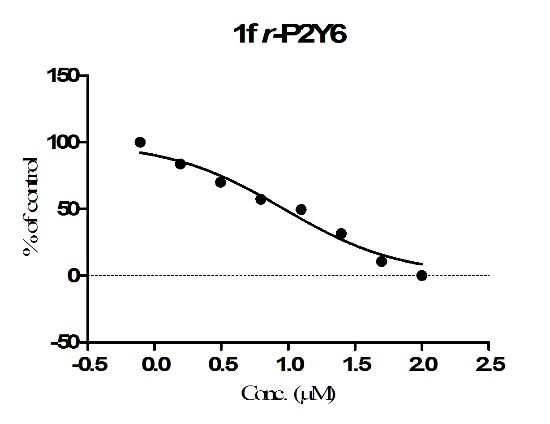** |
| **1g** | 40% |  |
| **1h** | 34% |  |
| **1i** | 41% |  |
| **1j** | 25.37 ± 3.10 | **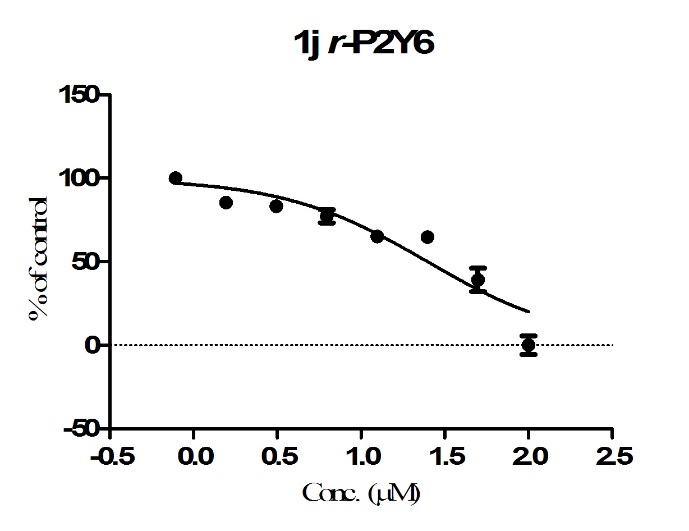** |
| **1k** | 8.49 ± 1.23 | **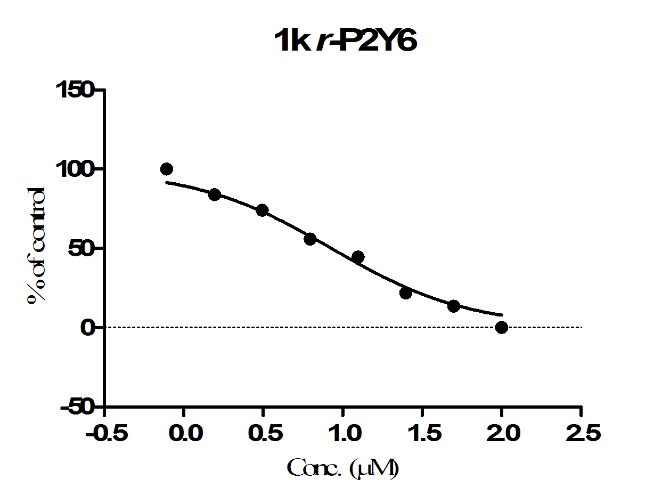** |
| **1l** | 43% |  |
| **1m** | 21% |  |
| **1n** | 7.06 ± 1.47 | **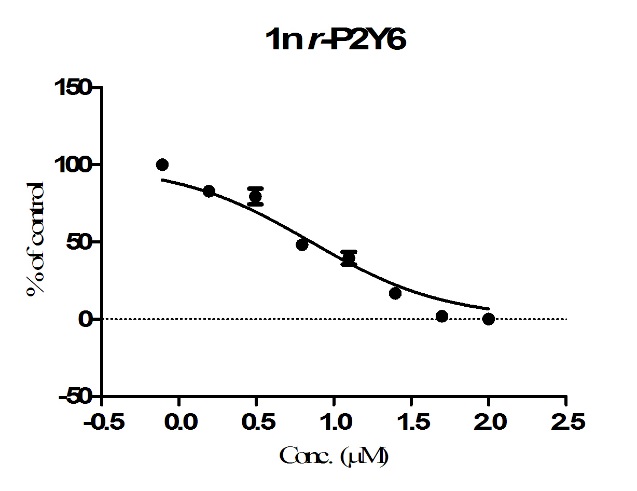** |
| **1o** | 32% |  |
| **1p** | 16.30 ± 0.83 | **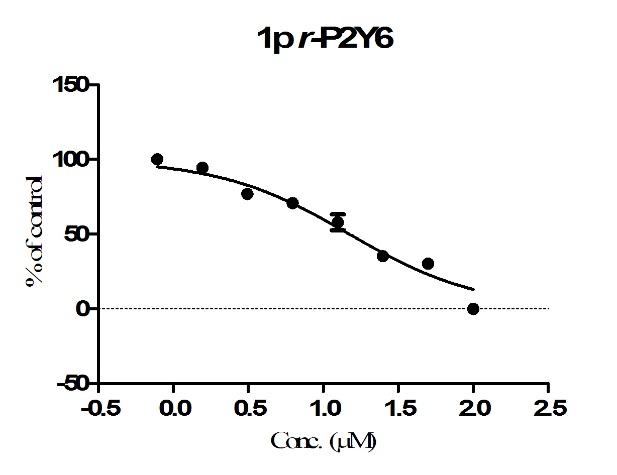** |
| **1q** | 48% |  |
| **1r** | 34% |  |
| **1s** | 12.68 ± 1.02 | **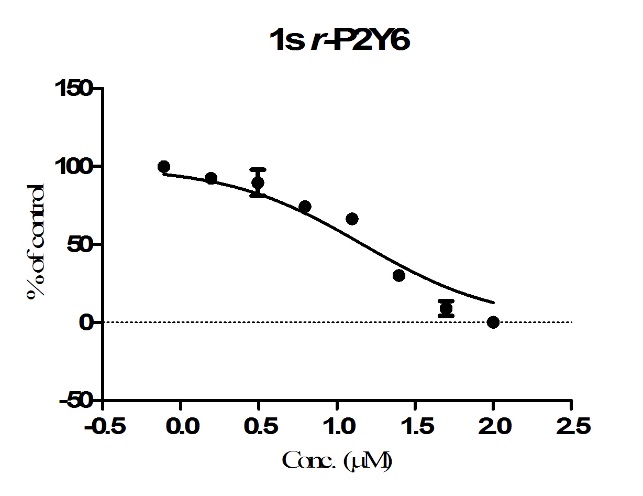** |
| **1t** | 12.36 ± 1.10 | **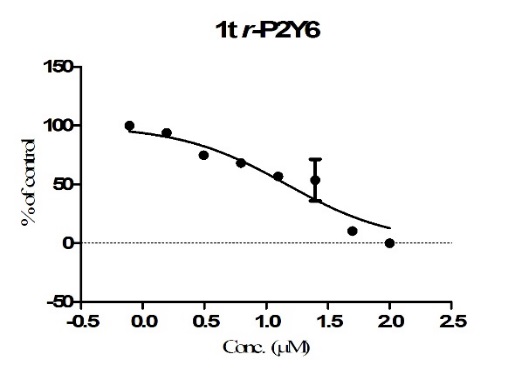** |
| **1u** | 18% |  |
| **1v** | 19% |  |
| **1w** | 39% |  |
| **1x** | 41% |  |
| **1y** | 23% |  |

# Supplementary Table 3: Proposed Pharmacokinetic and physicochemical properties of synthesized compounds (1a-1y)

| **Code** | **MW** | **H-bond acceptors** | **H-bond donors** | **TPSA** | **WLOGP** | **GI absorption** | **Lipinski violations** |
| --- | --- | --- | --- | --- | --- | --- | --- |
| 1a | 444.22 | 7 | 3 | 181.51 | 3.57 | Low | 0 |
| 1b | 375.36 | 6 | 2 | 161.28 | 2.87 | Low | 0 |
| 1c | 420.36 | 8 | 2 | 207.1 | 2.78 | Low | 1 |
| 1d | 406.33 | 8 | 2 | 207.1 | 2.47 | Low | 1 |
| 1e | 399.97 | 5 | 3 | 135.69 | 3.67 | Low | 0 |
| 1f | 350.78 | 4 | 2 | 115.46 | 3.31 | High | 0 |
| 1g | 375.36 | 6 | 2 | 161.28 | 2.87 | Low | 0 |
| 1h | 433.94 | 5 | 3 | 135.69 | 4.32 | Low | 0 |
| 1i | 385.23 | 4 | 2 | 115.46 | 3.96 | High | 0 |
| 1j | 409.81 | 6 | 2 | 161.28 | 3.52 | Low | 0 |
| 1k | 395.78 | 6 | 2 | 161.28 | 3.22 | Low | 0 |
| 1l | 453.51 | 4 | 1 | 89.44 | 6.4 | Low | 0 |
| 1m | 377.42 | 4 | 1 | 89.44 | 4.73 | High | 0 |
| 1n | 411.86 | 4 | 1 | 89.44 | 5.38 | High | 0 |
| 1o | 422.41 | 6 | 1 | 135.26 | 4.64 | Low | 0 |
| 1p | 365.79 | 5 | 1 | 98.67 | 3.73 | High | 0 |
| 1q | 335.77 | 4 | 1 | 89.44 | 3.72 | High | 0 |
| 1r | 411.86 | 4 | 1 | 89.44 | 5.38 | High | 0 |
| 1s | 368.22 | 4 | 1 | 87.22 | 3.04 | High | 0 |
| 1t | 380.76 | 6 | 1 | 135.26 | 3.63 | Low | 0 |
| 1u | 361.37 | 6 | 1 | 107.9 | 3.08 | High | 0 |
| 1v | 365.79 | 5 | 1 | 98.67 | 3.73 | High | 0 |
| 1w | 330.36 | 4 | 2 | 115.46 | 2.96 | High | 0 |
| 1x | 364.81 | 4 | 2 | 115.47 | 3.62 | High | 0 |
| 1y | 392.42 | 4 | 2 | 115.46 | 4.32 | High | 0 |

**
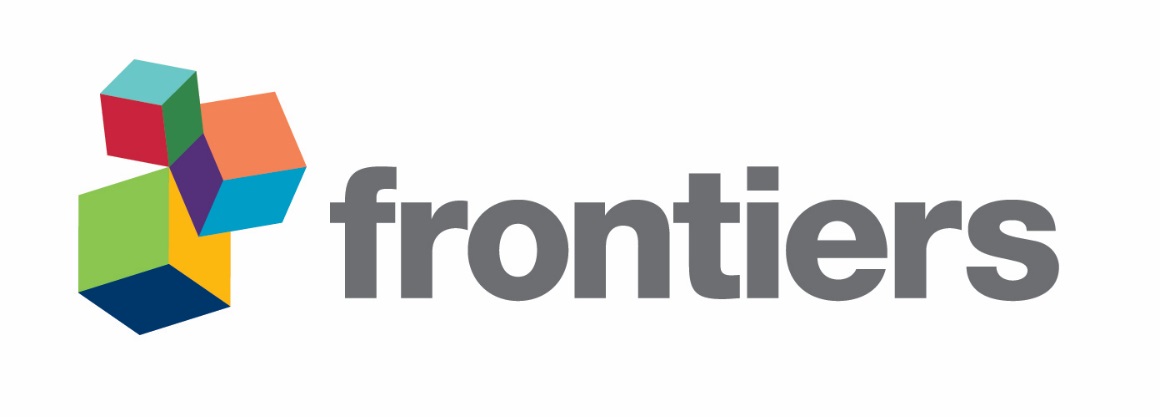
**
